# Wound healing outcomes in diabetic kidney disease patients receiving SGLT2 inhibitor therapy: a prospective propensity score-matched cohort study

**DOI:** 10.3389/fendo.2026.1793030

**Published:** 2026-06-03

**Authors:** Rong-rong Zhang, Siyue Huang, Xinjie Wang, Xiaoqin Li

**Affiliations:** Department of Nephrology, Xiangyang Central Hospital, Affiliated Hospital of Hubei University of Arts and Science, Xiangyang, Hubei, China

**Keywords:** diabetic foot ulcer, diabetic kidney disease, nephroprotection, propensity score matching, SGLT2 inhibitors, wound healing

## Abstract

**Background:**

The remarkable cardiorenal benefits of sodium-glucose cotransporter-2 (SGLT2) inhibitors have positioned these agents as cornerstone therapies for diabetic kidney disease (DKD). However, theoretical concerns regarding glycosuria-mediated effects on tissue perfusion have left clinicians uncertain about their safety in patients with active wounds. We conducted a prospective cohort study to characterize wound healing outcomes in this vulnerable population.

**Methods:**

Between January 2018 and December 2023, we prospectively enrolled 247 adults with type 2 diabetes mellitus, estimated glomerular filtration rate (eGFR) of 30–90 mL/min/1.73m², and wounds requiring medical attention at a tertiary wound care center. Participants receiving SGLT2 inhibitors (dapagliflozin, empagliflozin, or canagliflozin) for ≥30 days were matched 1:1 with unexposed controls using propensity scores derived from 15 baseline covariates. The primary endpoint was the time to complete epithelialization; secondary endpoints included wound infection and renal progression at six months.

**Results:**

Following propensity score matching, 204 patients (102 per cohort) with excellent covariate balance (all standardized mean differences <0.10) were analyzed. Wound healing trajectories were virtually identical between the cohorts: median healing time was 33 days (interquartile range [IQR]: 22–52) with SGLT2 inhibitors versus 34 days (24–62) in controls (adjusted hazard ratio [HR], 1.08; 95% confidence interval [CI], 0.82–1.42; p=0.594). No statistically significant difference in infection rates was observed (38.2% vs. 32.4%; unadjusted p=0.464; adjusted p=0.310). During the six-month follow-up period, no renal progression events were observed among SGLT2 inhibitor users (0% vs. 5.9%; p=0.029) and demonstrated superior eGFR preservation (−2.3 ± 3.8 vs. −5.5 ± 4.5 mL/min/1.73m²; p<0.001).

**Conclusions:**

No evidence of impaired wound healing or increased infection risk was observed with SGLT2 inhibitor therapy in patients with DKD; however, the anticipated nephroprotective benefits were preserved during the six-month follow-up period. These findings argue against the withholding of these beneficial agents in patients with concurrent wounds.

## 1 Introduction

Few therapeutic advances in diabetes care have generated as much enthusiasm—or controversy—as sodium-glucose cotransporter-2 (SGLT2) inhibitors. Within a decade of their introduction, these agents have fundamentally altered the treatment paradigm for type 2 diabetes, transitioning from glucose-lowering medications to disease-modifying therapies with profound cardiovascular and renal benefits extending beyond glycemic control (1, 2). The CREDENCE trial demonstrated that canagliflozin reduced the risk of kidney failure by 30% in patients with type 2 diabetes and albuminuric chronic kidney disease (3). The DAPA-CKD trial subsequently revealed that dapagliflozin conferred kidney and cardiovascular protection irrespective of diabetes status, with a hazard ratio of 0.61 for the primary composite outcome (4). Most recently, the EMPA-KIDNEY trial corroborated these salutary effects across an even more heterogeneous population (5). These landmark findings prompted the 2024 kidney disease: Improving Global Outcomes (KDIGO) guidelines to recommend SGLT2 inhibitors as first-line therapy for virtually all patients with diabetic kidney disease (DKD), a Grade 1A recommendation reflecting the highest level of evidence (6–8).

However, despite this compelling evidence, a substantial proportion of eligible patients never receive these life-altering medications. The most frequently cited reasons for withholding SGLT2 inhibitors are the presence of wounds, foot ulcers, or perceived amputation risk, concerns that trace their origins to an unexpected finding from the CANVAS Program published in 2017 (9). This trial reported a near doubling of the lower extremity amputation risk with canagliflozin (hazard ratio 1.97, 95% confidence interval 1.41–2.75), a signal that reverberated through the diabetes community and prompted regulatory safety warnings (10). Although subsequent large-scale trials failed to replicate this association, and the United States Food and Drug Administration ultimately removed the boxed warning from canagliflozin’s label in 2020 (11), the shadow of doubt has proven remarkably persistent.

The biological plausibility of SGLT2 inhibitor-mediated wound healing impairment is based on straightforward pharmacological principles. By blocking glucose reabsorption in the proximal tubule, these agents induce glycosuria exceeding 70 g daily, triggering osmotic diuresis and potential intravascular volume depletion (12). Reduced tissue perfusion, according to this argument, could compromise the delivery of oxygen, nutrients, and immune cells essential for wound repair. The glucose-rich urinary environment may additionally foster genitourinary infections with a theoretical risk of ascending soft tissue involvement (13). These mechanistic concerns have proven sufficiently persuasive, and many clinicians reflexively discontinue or avoid SGLT2 inhibitors in patients with diabetic foot ulcers, surgical wounds, or other integumentary compromises.

Patients with DKD are particularly susceptible to impaired wound healing owing to the convergence of multiple pathophysiological derangements (14). The uremic milieu is characterized by the accumulation of protein-bound and middle-molecule toxins that impair cellular proliferation, suppress angiogenesis, and attenuate the inflammatory cascade necessary for orderly tissue repair (15). Chronic low-grade inflammation, endothelial dysfunction, and accelerated atherosclerosis—hallmarks of advanced DKD—further compromise peripheral tissue perfusion and oxygen delivery to healing wounds (16). These systemic disturbances are compounded by the high prevalence of peripheral neuropathy, which diminishes protective sensation and predisposes to repetitive unrecognized trauma, and by hyperglycemia-mediated impairment of neutrophil chemotaxis, phagocytosis, and bactericidal activity (17). The resulting hostile microenvironment for tissue repair raises the question of whether SGLT2 inhibitors might further compromise wound healing in this population, particularly urgent and clinically consequential.

However, this practice pattern, however reasonable it may appear, carries substantial opportunity costs. Diabetic kidney disease affects 40% of individuals with type 2 diabetes and represents the leading cause of end-stage kidney disease worldwide (6–8, 18). Approximately 18.6 million people worldwide are affected by diabetic foot ulcers each year, with lifetime incidence rates of 19%–34% among individuals with diabetes (19). These ulcers precede 80% of lower extremity amputations and carry a five-year mortality rate exceeding 30%, which is higher than that of many common malignancies (20, 21). Recurrence after initial healing is distressingly common, with an estimated 42% within one year and 65% within five years (21). For patients with concomitant diabetic kidney disease, the imperative to optimize both wound healing and renal protection creates a therapeutic tension that current evidence inadequately addresses.

Existing evidence offers limited guidance. A recent comprehensive meta-analysis encompassing over 90,000 participants from large randomized trials concluded that the absolute benefits of SGLT2 inhibition outweigh the serious hazards of ketoacidosis or amputation (22). Furthermore, a Danish nationwide cohort study found no association between SGLT2 inhibitor use and an increased risk of lower limb amputation compared with sulfonylureas (23). However, no prospective investigation has systematically characterized wound healing trajectories in patients with DKD while simultaneously tracking renal function, a critical oversight given that the population for whom SGLT2 inhibitors offer the greatest benefit is also the population most susceptible to wound complications.

Therefore, we designed a prospective cohort study with the following objectives: the primary objective was to determine whether SGLT2 inhibitor therapy delays wound healing in patients with DKD and concurrent wounds; the co-primary objective was to evaluate whether SGLT2 inhibitor use increases the risk of wound infection; and the secondary objective was to assess whether the established nephroprotective benefits of these agents persist in this clinically complex population.

## 2 Materials and methods

### 2.1 Study design and oversight

We conducted a prospective observational cohort study at the Integrated Diabetes and Wound Care Center of Xiangyang Central Hospital, Affiliated Hospital of Hubei University of Arts and Science, a tertiary academic medical center that serves as a regional referral hub for complex diabetic wounds. The study protocol was approved by the Institutional Review Board prior to enrollment, and all participants provided written informed consent. An independent Data Safety Monitoring Board reviewed the accumulating data at prespecified intervals. The study adhered to the principles of the Declaration of Helsinki and was reported in accordance with the Strengthening the Reporting of Observational Studies in Epidemiology (STROBE) guidelines (24).

### 2.2 Participants

Between January 1, 2018, and December 31, 2023, we screened consecutive adults who presented to our wound care center for potential enrollment. The eligibility criteria were as follows: age ≥ 18 years; diagnosis of type 2 diabetes mellitus, confirmed by a medical record review; estimated glomerular filtration rate (eGFR) between 30 and 90 mL/min/1.73m², calculated using the Chronic Kidney Disease Epidemiology Collaboration equation (25); and the presence of a wound requiring professional medical management. Eligible wound types included diabetic foot ulcers, postoperative surgical incisions, traumatic lacerations, and other cutaneous lesions deemed by the treating physician to require structured wound care.

Participants were classified into the SGLT2 inhibitor cohort if they had been receiving dapagliflozin, empagliflozin, or canagliflozin continuously for at least 30 days prior to wound occurrence and maintained therapy throughout the observation period. The control cohort comprised patients with no SGLT2 inhibitor exposure in the 12 months preceding enrollment. We excluded individuals with type 1 diabetes, dialysis dependence, prior kidney transplantation, active malignancy requiring systemic therapy, chronic immunosuppressive medication use, wounds with exposed bone or joint capsule at presentation, and anticipated inability to attend scheduled follow-up visits.

### 2.3 Sample size determination

We estimated the required sample size based on published wound healing data indicating mean healing times of 45 ± 28 days for diabetic wounds of comparable severity (19, 26). To detect a clinically meaningful difference of 10 days between groups with 80% statistical power at a two-sided alpha of 0.05, we calculated that 98 participants per cohort would be necessary. The power calculation assumed equal variances between groups, which is the conventional approach when planning studies without prior group-specific variance estimates. *Post hoc* verification using Welch’s t-test framework under the observed variance structure (standard deviation [SD] = 22.2 in the SGLT2 inhibitor cohort; SD = 28.8 in the control cohort) confirmed that the achieved sample of 102 participants per cohort provided approximately 85% power, exceeding the originally targeted 80% threshold. Anticipating approximately 10% incomplete follow-up and the need for trimming during propensity score matching, we targeted the enrollment of 120 participants in each exposure category.

### 2.4 Baseline assessment

At enrollment, trained research coordinators collected comprehensive baseline data using standardized electronic case report forms. Demographic variables included age and self-reported sex. Clinical characteristics included diabetes duration, body mass index, most recent glycated hemoglobin (HbA1c) level within 90 days, serum creatinine level with calculated eGFR, spot urine albumin-to-creatinine ratio (UACR), and resting systolic blood pressure. Smoking status was categorized as current, former, or never smoked. The comorbidities of interest included peripheral vascular disease (defined as an ankle-brachial index < 0.90, prior lower extremity revascularization, or documented peripheral arterial disease) and diabetic peripheral neuropathy (defined as loss of protective sensation on a standardized 10-g monofilament examination). Medication data included SGLT2 inhibitor type and treatment duration, as well as concurrent use of renin-angiotensin system inhibitors and statins.

The wound characteristics recorded at baseline included anatomical location, wound type (diabetic foot ulcer, surgical incision, traumatic wound, or other), wound dimensions measured as the maximum length multiplied by the maximum perpendicular width in square centimeters, and the presence of clinical signs of infection. For diabetic foot ulcers, we additionally documented the University of Texas classification grade and stages (27).

### 2.5 Propensity score estimation and matching

**Figure 1:**
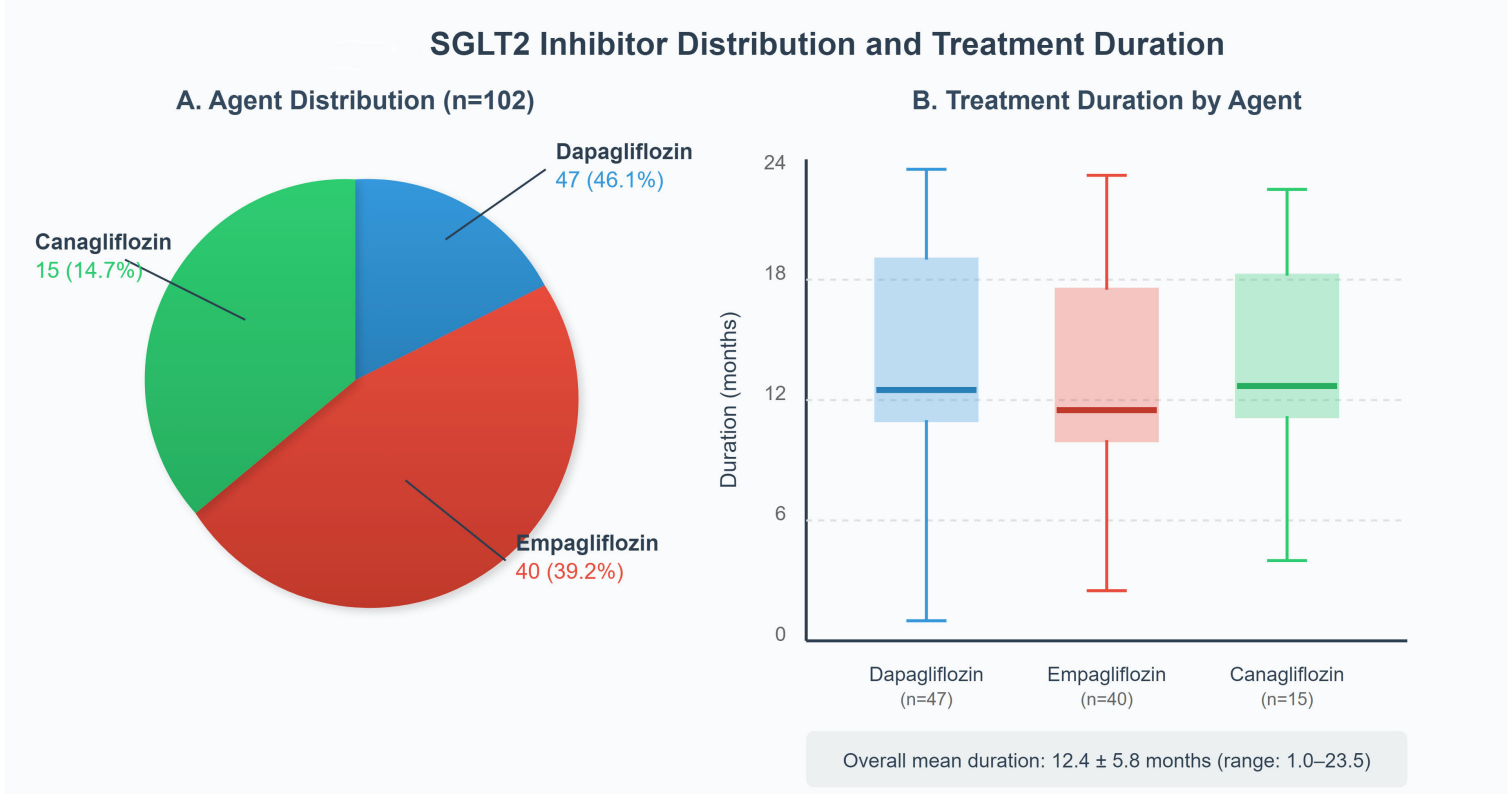
SGLT2 inhibitor distribution and treatment durations. **(A)** Pie chart illustrating the distribution of SGLT2 inhibitor agents among the 102 participants in the matched cohort. Dapagliflozin was the most frequently prescribed agent (n=47, 46.1%), followed by empagliflozin (n=40, 39.2%) and canagliflozin (n=15, 14.7%). **(B)** Box-and-whisker plots depicting the treatment duration (months) with SGLT2 inhibitor agents. Horizontal lines within boxes represent medians; box boundaries represent interquartile ranges; whiskers extend to the minimum and maximum values. The overall mean treatment duration was 12.4 ± 5.8 months (range 1.0–23.5 months), indicating that participants were receiving established therapy rather than recently initiated treatment at the time of wound occurrence.

To address confounding by indication inherent in observational treatment comparisons, we employed propensity score matching to create balanced cohorts24. The propensity score, which is the conditional probability of receiving SGLT2 inhibitor therapy given the observed baseline characteristics, was estimated using multivariable logistic regression incorporating 15 covariates selected based on clinical relevance and demonstrated associations with both treatment selection and wound outcomes: age, sex, body mass index, diabetes duration, HbA1c, baseline eGFR, log-transformed UACR, systolic blood pressure, smoking status, peripheral vascular disease, diabetic neuropathy, wound type, wound size, angiotensin-converting enzyme inhibitor or angiotensin receptor blocker use, and statin use (**Supplementary Methods S1**; **Supplementary Table 1**). SGLT2 inhibitor type, dosage, and duration of therapy were intentionally excluded from the propensity score model because these variables represent attributes of the treatment exposure itself rather than pre-treatment confounders; their inclusion could introduce collider bias and is conceptually inappropriate for propensity score estimation. These treatment-specific characteristics are presented descriptively in **Table 1** and **Figure 1**.

**Table 1:** Baseline characteristics following propensity score matching.

Characteristic	SGLT2 inhibitor (n=102)	Control (n=102)	SMD
Demographics			
Age, years	58.4 ± 9.9	56.3 ± 9.0	0.08
Male sex, n (%)	55 (53.9)	54 (52.9)	0.02
Clinical parameters			
Body mass index, kg/m²	27.3 ± 3.5	28.9 ± 4.2	0.09
Diabetes duration, years	13.6 ± 7.0	12.2 ± 6.1	0.08
Glycated hemoglobin, %	7.8 ± 1.1	8.1 ± 1.1	0.09
eGFR, mL/min/1.73m²	57.4 ± 14.5	54.6 ± 15.3	0.09
UACR, mg/g	533 ± 701	545 ± 624	0.02
Systolic blood pressure, mmHg	137.9 ± 17.9	134.4 ± 16.4	0.07
Smoking status, n (%)			
Current	15 (14.7)	16 (15.7)	0.03
Former	34 (33.3)	32 (31.4)	0.04
Never	53 (52.0)	54 (52.9)	0.02
Comorbidities, n (%)			
Peripheral vascular disease	39 (38.2)	27 (26.5)	0.08
Diabetic neuropathy	46 (45.1)	43 (42.2)	0.06
Concomitant medications, n (%)			
ACE inhibitor or ARB	84 (82.4)	92 (90.2)	0.09
Statin	75 (73.5)	77 (75.5)	0.05
Wound characteristics			
Wound size, cm²	4.9 ± 3.4	5.0 ± 4.2	0.03
Wound type, n (%)			
Diabetic foot ulcer	43 (42.2)	44 (43.1)	0.02
Surgical incision	32 (31.4)	28 (27.5)	0.08
Traumatic wound	22 (21.6)	22 (21.6)	0.00
Other	5 (4.9)	8 (7.8)	0.07

Data are presented as mean ± standard deviation or n (%).

ACE, angiotensin-converting enzyme; ARB, angiotensin receptor blocker; eGFR, estimated glomerular filtration rate; SMD, standardized mean difference; UACR, urine albumin-to-creatinine ratio.

SMD values <0.10 indicate negligible imbalance between groups.

We implemented 1:1 nearest-neighbor matching without replacement using a caliper width of 0.20 standard deviations of the propensity score logit, a threshold empirically shown to eliminate approximately 98% of the bias from measured confounders (28). The adequacy of covariate balance following matching was assessed by calculating the standardized mean differences for all baseline variables, with absolute values below 0.10 considered indicative of negligible imbalance (29). We also evaluated the propensity score overlap between cohorts through visual inspection of the distribution histograms, and confirmed that all matched pairs fell within the region of common support (**Supplementary Methods S2**; **Supplementary Figure 1**).

### 2.6 Follow-up procedures

The participants underwent a structured follow-up according to a standardized protocol. Wound assessments were performed weekly for active diabetic foot ulcers and biweekly for surgical and traumatic wounds, with the visit frequency adjusted based on the clinical trajectory at the discretion of the treating physician. At each encounter, a wound care specialist measured wound dimensions using calibrated digital planimetry, documented the healing status, assessed for clinical signs of infection, and recorded any adverse events. Complete wound healing was defined as full epithelialization with no drainage, independently confirmed by a second wound care specialist blinded to the SGLT2 inhibitor exposure status.

Laboratory assessments, including serum creatinine for eGFR calculation, were performed at baseline, three months, and six months. The observation period for each participant was extended until complete wound healing or 180 days after enrollment, whichever occurred first.

### 2.7 Outcome measures

The primary endpoint was defined as the time from enrollment to complete wound healing. The prespecified secondary endpoints included wound infection requiring systemic antibiotic therapy, classified according to the Infectious Diseases Society of America criteria as superficial, deep soft tissue, or osteomyelitis (30); renal progression, defined as a sustained reduction in eGFR exceeding 30% from baseline confirmed on two consecutive measurements separated by at least 28 days; and absolute change in eGFR from baseline to six months.

### 2.8 Statistical analysis

Continuous variables were assessed for normal distribution using the Shapiro–Wilk test (**Supplementary Table 3**). Variables approximating normal distributions are presented as means ± standard deviation and were compared using an independent sample t-test. Non-normally distributed variables are presented as medians with interquartile ranges and were compared using the Mann–Whitney U test. Categorical variables are expressed as frequencies with percentages and were compared using the chi-square test or Fisher’s exact test, as appropriate.

Time to wound healing was analyzed using Kaplan–Meier estimation with between-group comparisons using the log-rank test. We constructed multivariable Cox proportional hazards models to estimate adjusted hazard ratios with 95% confidence intervals, incorporating covariates with residual post-matching imbalance and clinically important prognostic factors. The proportional hazards assumption was evaluated by examining Schoenfeld residuals and visually inspecting log-log survival plots (**Supplementary Methods S3**; **Supplementary Figure 2**) (31).

For the wound infection endpoint, we fitted multivariable logistic regression models to estimate adjusted odds ratios and 95% confidence intervals. Continuous predictor variables in the logistic regression model were standardized to a mean of zero and a standard deviation of one prior to model fitting; accordingly, the reported odds ratios reflect the change in infection odds per one standard deviation increase in each continuous predictor. Model discrimination was quantified using the concordance statistic, and calibration was assessed using the Hosmer-Lemeshow goodness-of-fit test (**Supplementary Figure 3**). Renal disease progression was evaluated during the 6-month follow-up. Time-to-event analyses were assessed using Cox proportional hazards regression, and given the low event rate, the six-month event comparison was additionally tested using Fisher’s exact test.

We conducted several prespecified sensitivity analyses to evaluate the robustness of the findings: inverse probability of treatment weighting as an alternative to matching (**Supplementary Methods S6**), calculation of E-values to quantify the strength of unmeasured confounding necessary to explain away observed associations (**Supplementary Methods S5**) (32), and multiple imputation using chained equations for participants with incomplete covariate data (**Supplementary Methods S4**). Subgroup analyses examined wound healing according to wound type, baseline eGFR stratum, glycemic control category, and peripheral vascular disease status, with interaction terms included in the regression models to test for effect modification.

All hypothesis tests were two-sided, with a significance threshold of 0.05. Analyses were performed using Python version 3.10 with the SciPy (v1.9.3), statsmodels (v0.13.5), and lifelines (v0.27.4) packages (**Supplementary Methods S7**).

## 3 Results

### 3.1 Participant flow and baseline characteristics

**Figure 2:**
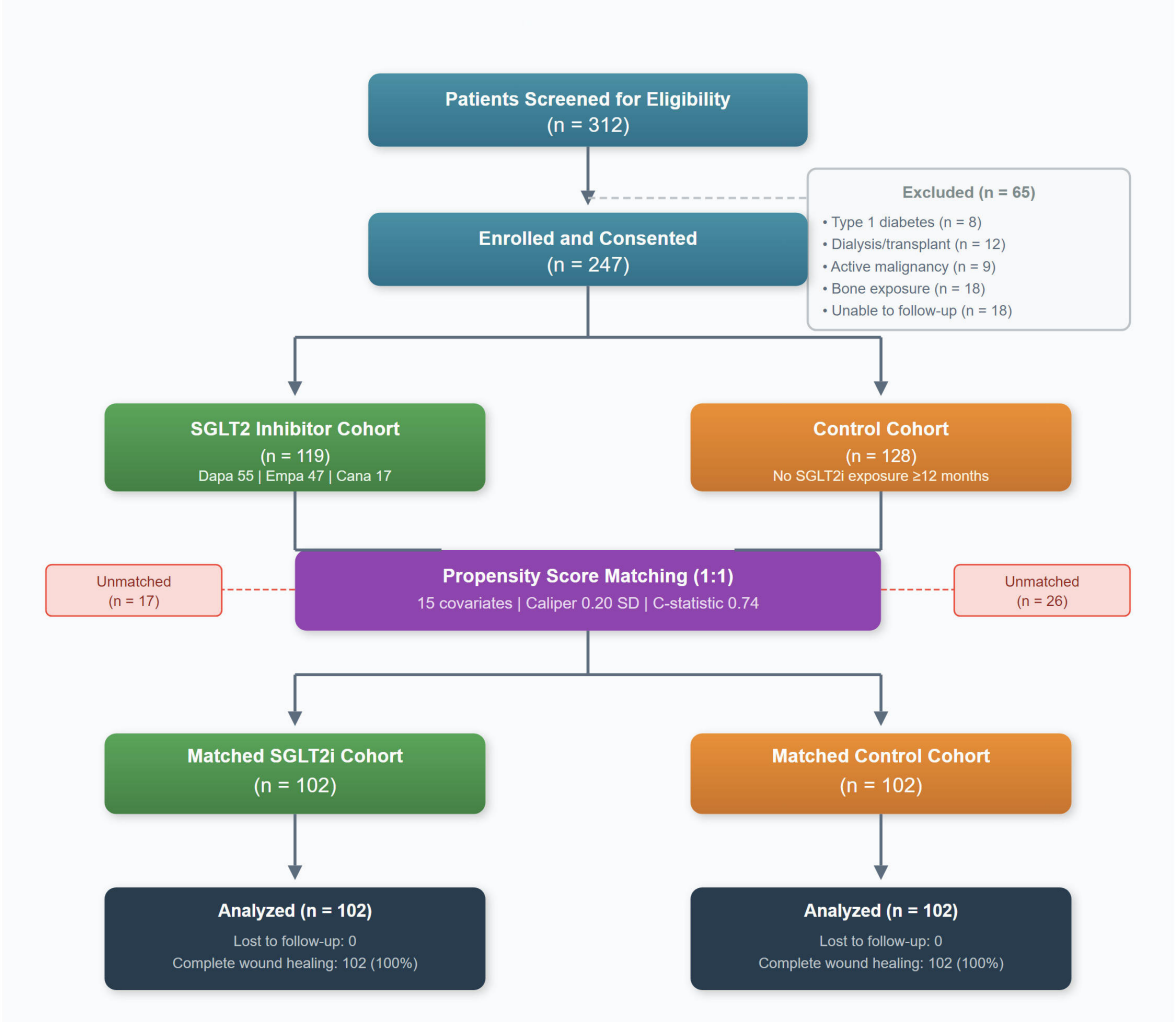
Study flow diagram. CONSORT-style flow diagram depicting participant screening, enrollment, allocation to exposure cohorts, propensity score matching, and final analysis. Of the 312 patients screened for eligibility, 65 were excluded (type 1 diabetes, n=8; dialysis/transplant, n=12; active malignancy, n=9; bone exposure, n=18; unable to follow-up, n=18), leaving 247 enrolled participants (SGLT2 inhibitor cohort, n=119; control cohort, n=128). Following 1:1 nearest-neighbor propensity score matching with a caliper of 0.20 standard deviations (15 covariates; C-statistic 0.74), 17 SGLT2 inhibitor users and 26 controls remained unmatched in the study. The final analysis included 102 matched pairs (204 participants). No participants were lost to follow-up, and complete wound healing was achieved in 100% of both cohorts within the 180-day observation period.

Over the 6-year enrollment period, we screened 312 patients for eligibility, of whom 247 met the inclusion criteria and provided informed consent: 119 received SGLT2 inhibitor therapy and 128 had no exposure (**Figure 2**). Following propensity score matching, 102 well-balanced pairs (204 participants) were retained for analysis. No participants were lost to follow-up before achieving the primary endpoint or completing the 180-day observation period, ensuring complete outcome assessment.

**Figure 3:**
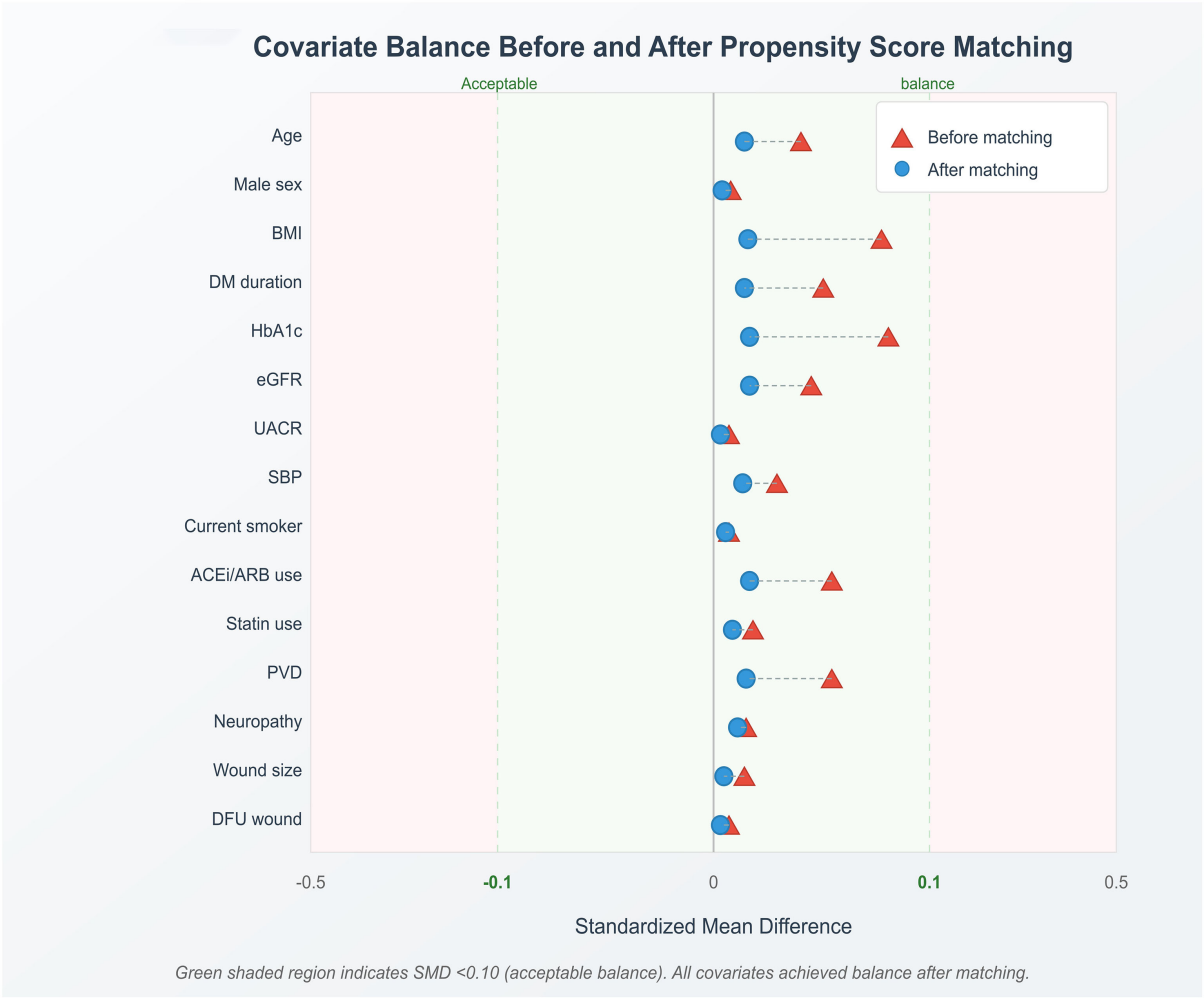
Covariate balance before and after propensity score matching. Love plot displaying standardized mean differences (SMD) for all 15 baseline covariates included in the propensity score model. Red triangles represent SMD values before matching, and blue circles represent SMD values after matching. The green-shaded region demarcates the zone of acceptable balance (absolute SMD <0.10). Prior to matching, substantial imbalances were observed in body mass index (SMD 0.42), HbA1c (SMD 0.43), diabetes duration (SMD 0.27), eGFR (SMD 0.24), peripheral vascular disease (SMD 0.30), and ACE inhibitor/ARB use (SMD 0.29). Propensity score matching achieved excellent covariate balance, with all post-matching SMD values falling below the 0.10 threshold, indicating a negligible residual imbalance across all measured confounders.

The propensity score model demonstrated good discriminatory capacity (C-statistic 0.74, 95% confidence interval 0.68–0.80). Before matching, notable imbalances existed between the cohorts: SGLT2 inhibitor users had a lower body mass index (standardized mean difference 0.42), better glycemic control (standardized mean difference 0.43 for HbA1c), and a higher prevalence of peripheral vascular disease (standardized mean difference 0.30), a pattern consistent with channeling bias, wherein clinicians may preferentially prescribe these agents to patients perceived as having favorable risk-benefit profiles despite coexisting vascular disease. Propensity score matching successfully eliminated these imbalances; all standardized mean differences fell below 0.10 following matching, indicating excellent covariate balance across the 15 measured confounders (**Table 1**, **Figure 3**, **Supplementary Table 2**).

The matched cohorts comprised middle-aged adults (mean age 58.4 ± 9.9 years in the SGLT2 inhibitor group vs. 56.3 ± 9.0 years in the control group) with a slight male predominance (53.9% vs. 52.9%). Participants had longstanding diabetes (mean duration 13.6 ± 7.0 vs. 12.2 ± 6.1 years) with moderately impaired kidney function (mean eGFR 57.4 ± 14.5 vs. 54.6 ± 15.3 mL/min/1.73m²) and substantial albuminuria (mean UACR 533 ± 701 vs. 545 ± 624 mg/g). Glycemic control was suboptimal in both groups (mean HbA1c 7.8 ± 1.1% vs. 8.1 ± 1.1%). Diabetic foot ulcers were the most common wound type (42.2% vs. 43.1%), followed by surgical incisions (31.4% vs. 27.5%) and traumatic wounds (21.6% vs. 21.6%). The mean wound size was comparable between the groups (4.9 ± 3.4 vs. 5.0 ± 4.2 cm²).

Among SGLT2 inhibitor users, dapagliflozin was the most commonly prescribed agent (47 patients, 46.1%), followed by empagliflozin (40 patients, 39.2%) and canagliflozin (15 patients, 14.7%). The mean treatment duration prior to wound occurrence was 12.4 ± 5.8 months, indicating established therapy rather than recent initiation (**Figure 1** and **Supplementary Table 7**).

### 3.2 Primary endpoint: time to wound healing

All 204 participants achieved complete wound healing within the 180-day observation period (**Table 2**). No wounds remained unhealed at the conclusion of the follow-up, and no participants were censored for non-healing. The primary analysis revealed no statistically significant differences in healing trajectories between the cohorts. The median time to complete epithelialization was 33 days (interquartile range [IQR]: 22–52) in SGLT2 inhibitor users compared with 34 days (IQR: 24–62) in controls. The mean healing time was numerically shorter with SGLT2 inhibitor therapy (39.8 ± 22.2 vs. 44.6 ± 28.8 days), representing an absolute difference of 4.8 days and a relative reduction of 10.8%; however, this difference was not statistically significant (t-test: degrees of freedom =202, p=0.183; Mann–Whitney U test: n=(102, 102), p=0.267).

**Table 2:** Wound healing outcomes by treatment group.

Outcome	SGLT2 inhibitor (n=102)	Control (n=102)	Effect estimate (95% CI)	p-value
Primary endpoint				
Complete wound healing, n (%)	102 (100)	102 (100)	—	—
Time to healing, days				
Mean ± SD	39.8 ± 22.2	44.6 ± 28.8	MD −4.8 (−11.8 to 2.2)	0.183^a^
Median (IQR)	33 (22–52)	34 (24–62)	—	0.267^b^
Adjusted HR for healing	—	—	1.08 (0.82–1.42)	0.594^c^
Healing by time point, n (%)				
Day 30	63 (61.8)	60 (58.8)	RR 1.05 (0.84–1.31)	0.661
Day 60	90 (88.2)	87 (85.3)	RR 1.03 (0.94–1.14)	0.544
Day 90	99 (97.1)	96 (94.1)	RR 1.03 (0.98–1.09)	0.317
Secondary endpoints				
Wound infection, n (%)	39 (38.2)	33 (32.4)	RR 1.18 (0.82–1.70)	0.464^d^
Adjusted OR for infection	—	—	1.20 (0.89–1.69)	0.310^e^
Renal progression, n (%)	0 (0)	6 (5.9)	ARR 5.9% (1.1–10.7)	0.029^f^
eGFR change, mL/min/1.73m²	−2.3 ± 3.8	−5.5 ± 4.5	MD 3.2 (2.0–4.4)	<0.001^a^

ARR, absolute risk reduction; CI, confidence interval; eGFR, estimated glomerular filtration rate; HR, hazard ratio; IQR, interquartile range; MD, mean difference; OR, odds ratio; RR, relative risk; SD, standard deviation; SGLT2, sodium-glucose cotransporter-2.

^a^Independent samples t-test (df = 202). ^b^ Mann-Whitney U test. ^c^ Multivariable Cox proportional hazards regression adjusted for wound size, glycated hemoglobin, and peripheral vascular disease. ^d^ Chi-square test (df = 1). ^e^ Multivariable logistic regression adjusted for covariates listed in **Table 4**. ^f^ Fisher’s exact test.

**Figure 4:**
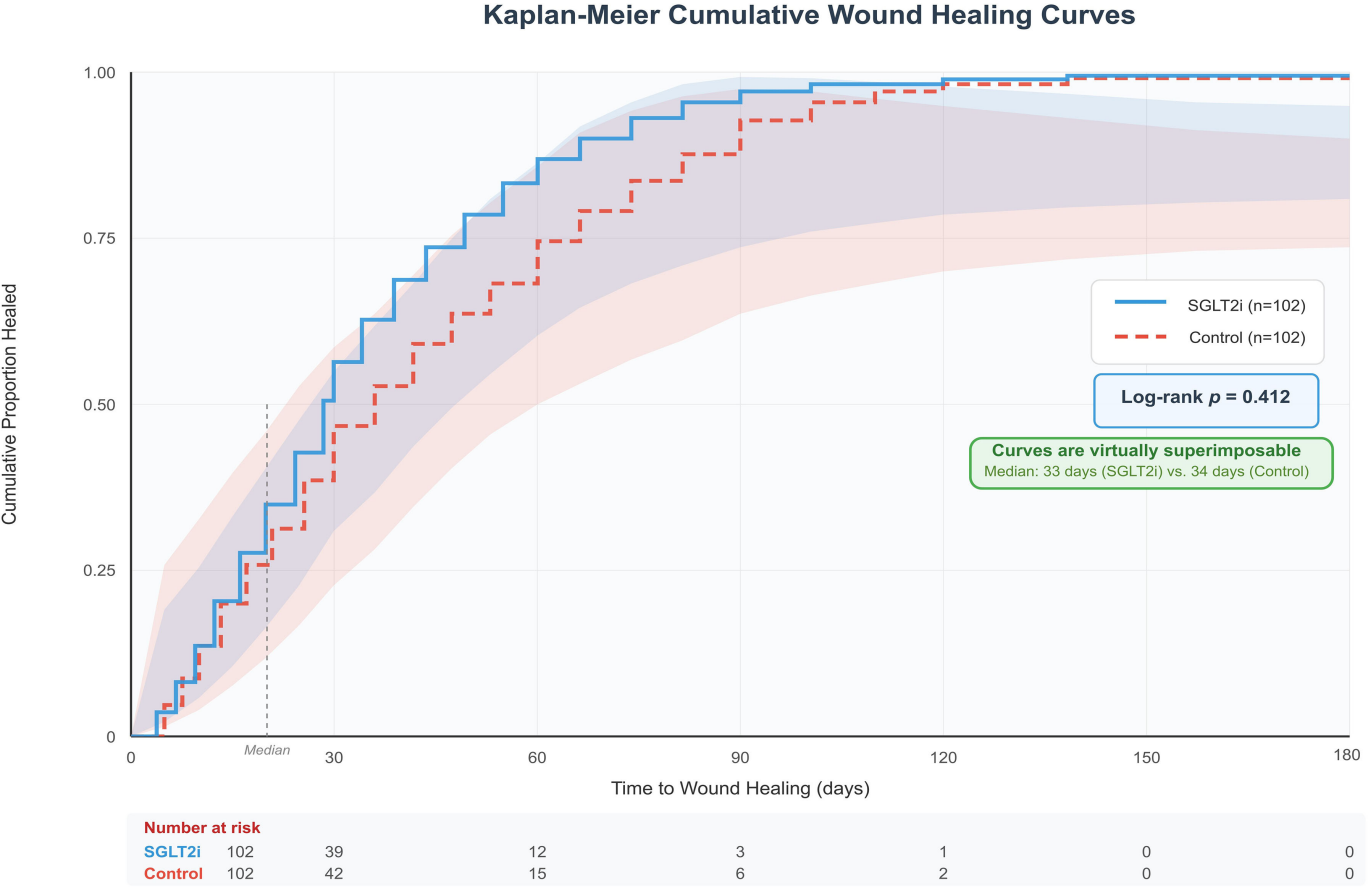
Kaplan-Meier cumulative wound healing curves. Time-to-event analysis depicting the cumulative proportion of participants who achieved complete wound healing over the 180-day observation period. The solid blue line represents the SGLT2 inhibitor cohort (n=102), and the dashed red line represents the control cohort (n=102). The shaded regions denote the 95% confidence intervals. The vertical dashed gray line indicates the median healing time of the group. The healing curves are virtually superimposable between the cohorts (log-rank p=0.412). The median time to complete epithelialization was 33 days (IQR: 22–52) in SGLT2 inhibitor users versus 34 days (IQR: 24–62) in controls. The number at risk is displayed below the x-axis at 30-day intervals. Note: As depicted, the two healing curves are virtually superimposable throughout the entire observation period, confirming that SGLT2 inhibitor therapy was not associated with any detectable delay in wound healing. The log-rank p-value of 0.412 is annotated directly on the figure to facilitate immediate visual interpretation.

Kaplan–Meier analysis demonstrated virtually superimposable cumulative healing curves throughout the observation period (log-rank p=0.412; **Figure 4**). At 30 days, 63 (61.8%) in the SGLT2 inhibitor cohort and 60 (58.8%) participants in the control cohort achieved complete healing. By day 60, these proportions increased to 90 (88.2%) and 87 (85.3%), respectively. By day 90, healing was complete in 99 (97.1%) and 96 (94.1%) participants, respectively.

Multivariable Cox proportional hazards regression confirmed the absence of any significant association between SGLT2 inhibitor use and wound healing (**Table 3**). After adjusting for wound size, HbA1c, and peripheral vascular disease status, the hazard ratio for healing with SGLT2 inhibitor therapy was 1.08 (95% confidence interval 0.82–1.42; p=0.594), indicating neither acceleration nor impairment of the healing process. Wound size emerged as the strongest predictor of healing time (adjusted hazard ratio 0.95 per cm² increase, 95% confidence interval 0.91–0.99; p=0.008), with larger wounds requiring proportionally longer to epithelialize. A supplementary fully adjusted Cox model incorporating eGFR, UACR, and diabetic neuropathy as additional covariates yielded consistent results (adjusted HR: 1.06; 95% CI: 0.80–1.41; p = 0.672).

**Table 3:** Cox proportional hazards regression for time to wound healing.

Variable	Unadjusted HR (95% CI)	p-value	Adjusted HR (95% CI)	p-value
SGLT2 inhibitor use	1.12 (0.84–1.49)	0.412	1.08 (0.82–1.42)	0.594
Wound size, per cm²	0.94 (0.91–0.98)	0.003	0.95 (0.91–0.99)	0.008
Glycated hemoglobin, per %	0.89 (0.79–1.00)	0.052	0.91 (0.81–1.03)	0.127
Peripheral vascular disease	0.78 (0.58–1.05)	0.098	0.82 (0.61–1.11)	0.194

CI, confidence interval; HR, hazard ratio; SGLT2, sodium-glucose cotransporter-2.

Hazard ratios greater than 1.0 indicate faster wound healing. The adjusted model included wound size, glycated hemoglobin, and peripheral vascular disease status as covariates. The proportional hazards assumption was confirmed by Schoenfeld residual testing (global test: χ² = 0.42, p = 0.517) and visual inspection of log-log survival plots (**Supplementary Figure 2**).

### 3.3 Secondary endpoint: wound infection

**Figure 5:**
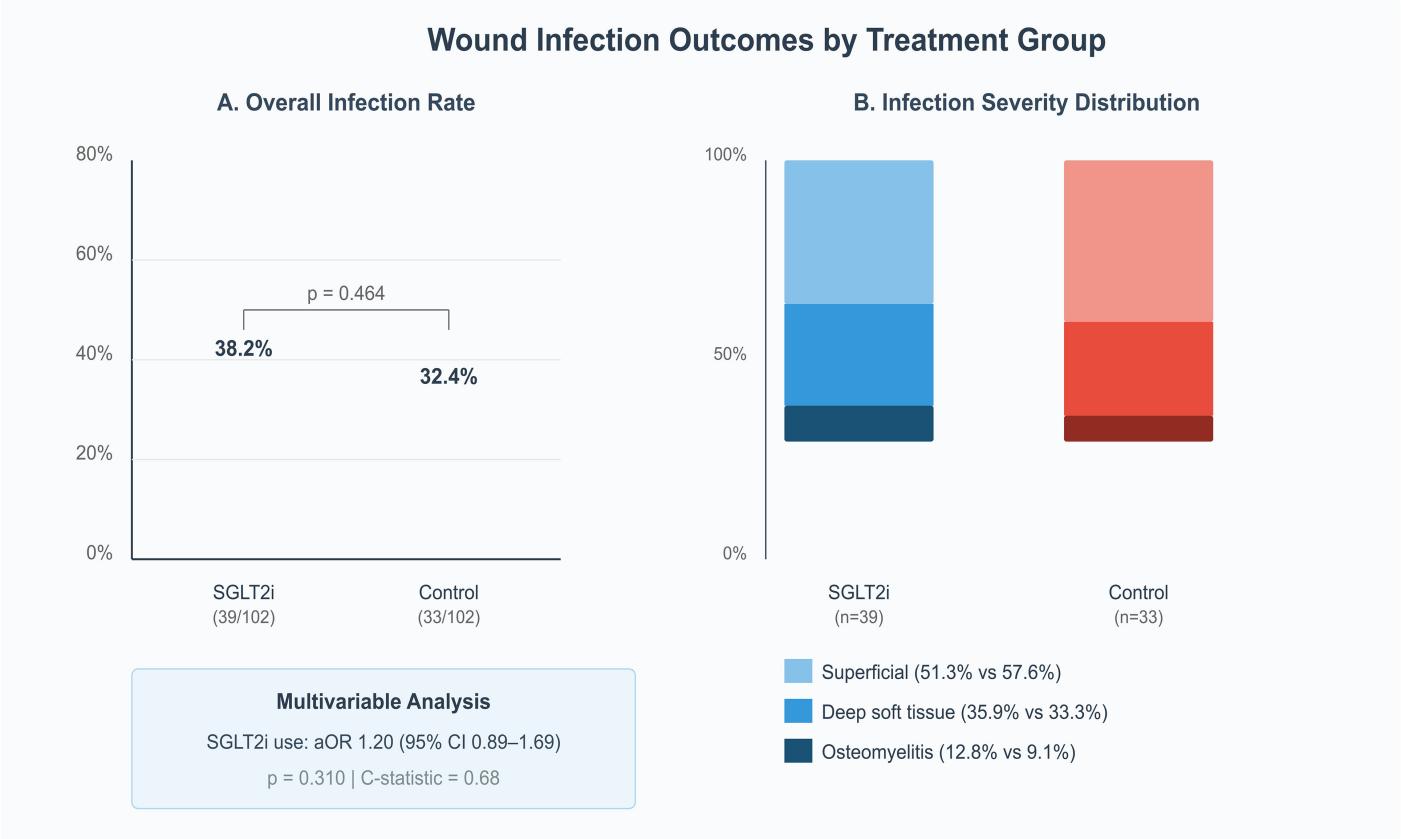
Wound infection outcomes by the treatment group. **(A)** Bar chart comparing overall wound infection rates requiring systemic antibiotic therapy between SGLT2 inhibitor users (38.2%, 39/102) and controls (32.4%, 33/102). The between-group difference was not statistically significant (χ² test: df=1, p=0.464). Error bars represent the 95% confidence interval. **(B)** Stacked bar chart depicting the distribution of infection severity among infected participants according to the Infectious Diseases Society of America classification. The severity distribution was similar between the groups (p =0.821): superficial infections (SGLT2i, 51.3% vs. control, 57.6%), deep soft tissue infections (35.9% vs. 33.3%), and osteomyelitis (12.8% vs. 9.1%). Multivariable logistic regression yielded an adjusted odds ratio of 1.20 (95% confidence interval 0.89–1.69; p=0.310) for wound infection with SGLT2 inhibitor use.

Wound infections requiring systemic antibiotic therapy occurred in 72 participants (35.3%): 39 (38.2%) in the SGLT2 inhibitor cohort and 33 (32.4%) in the control cohort (**Table 2**). No statistically significant difference was observed (relative risk 1.18, 95% confidence interval 0.82–1.70; χ² test: df=1, p=0.464). The distribution of infection severity was similar between the groups: superficial infections predominated in both cohorts (51.3% vs. 57.6% of infected wounds), followed by deep soft tissue infections (35.9% vs. 33.3%), and osteomyelitis (12.8% vs. 9.1%; p=0.821 for distribution comparison) (**Figure 5**).

In the multivariable analysis (**Table 4**), SGLT2 inhibitor use demonstrated no independent association with infection risk (adjusted odds ratio 1.20, 95% confidence interval 0.89–1.69; p=0.310). Poor glycemic control emerged as the strongest modifiable predictor; each 1-SD increase in HbA1c (approximately 1.1 percentage points, given the sample mean of 7.95% and SD of 1.1%) was associated with 48% higher infection odds (aOR, 1.48; 95% CI, 1.10–2.22; p=0.018). Diabetic neuropathy independently predicted infection (aOR, 1.39; 95% CI, 1.06–1.96; p=0.032), likely reflecting impaired protective sensation and unrecognized repetitive trauma. The logistic model demonstrated acceptable discrimination (C-statistic/AUC, 0.71) and good calibration (Hosmer–Lemeshow p=0.554; **Supplementary Figure 3**).

**Table 4:** Multivariable logistic regression for wound infection.

Variable	Adjusted OR (95% CI)	p-value
SGLT2 inhibitor use	1.20 (0.89–1.69)	0.310
Age, per SD	1.06 (0.76–1.44)	0.720
Body mass index, per SD	0.95 (0.68–1.32)	0.760
eGFR, per SD	0.98 (0.70–1.34)	0.850
Glycated hemoglobin, per SD	1.48 (1.10–2.22)	0.018
Wound size, per SD	0.90 (0.62–1.25)	0.480
Peripheral vascular disease	1.10 (0.79–1.52)	0.560
Diabetic neuropathy	1.39 (1.06–1.96)	0.032
ACE inhibitor or ARB use	1.20 (0.90–1.74)	0.280

ACE, angiotensin-converting enzyme; ARB, angiotensin receptor blocker; CI, confidence interval; eGFR, estimated glomerular filtration rate; OR, odds ratio; SD, standard deviation; SGLT2, sodium-glucose cotransporter-2.

Continuous variables were standardized (mean = 0, SD = 1) prior to model fitting. For HbA1c, the sample mean was 7.95% with a standard deviation of 1.1 percentage points; thus, the odds ratio of 1.48 reflects the increase in infection odds per approximately 1.1 percentage-point increment in HbA1c. Model performance: C-statistic (AUC) 0.71; Hosmer-Lemeshow goodness-of-fit test χ² = 6.84, df = 8, p = 0.554; Brier score 0.18. Calibration plot presented in **Supplementary Figure 3**.

### 3.4 Secondary endpoint: renal outcomes

**Figure 6:**
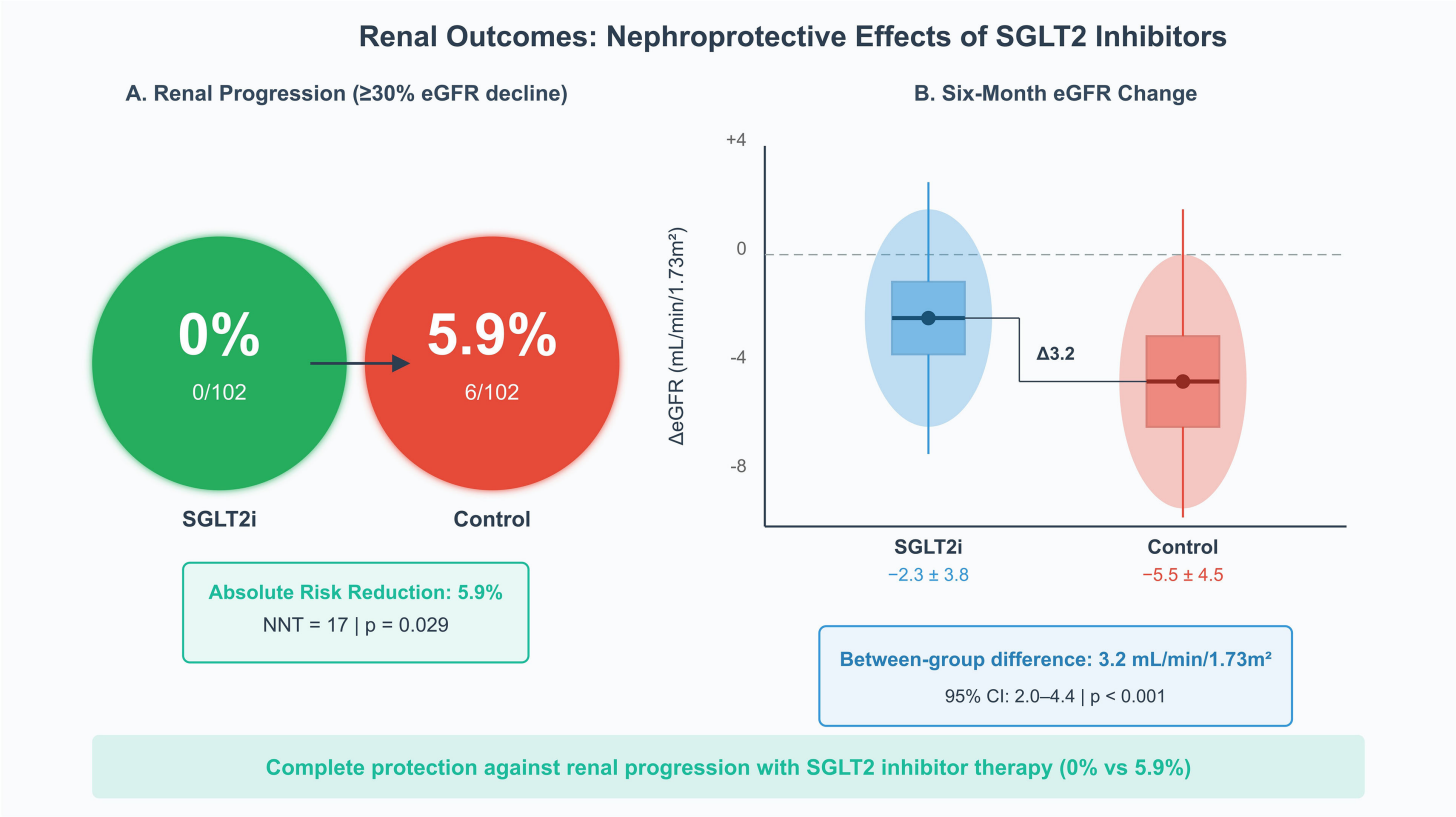
Renal outcomes: nephroprotective effects of SGLT2 inhibitors. **(A)** Proportional area chart illustrating renal progression rates, defined as a sustained eGFR decline exceeding 30% from baseline, confirmed by two consecutive measurements at least 28 days apart. No renal progression events were observed among SGLT2 inhibitor users (0/102, 0%) compared with controls (6/102, 5.9%; Fisher’s exact p=0.029). The absolute risk reduction of 5.9% corresponds to a number needed to treat of 17 patients. **(B)** Box-and-whisker plots with individual data points depicting the six-month change in eGFR (mL/min/1.73m²) by treatment group. SGLT2 inhibitor users demonstrated significantly attenuated eGFR decline (−2.3 ± 3.8 mL/min/1.73m²) compared with controls (−5.5 ± 4.5 mL/min/1.73m²), yielding a between-group difference of 3.2 mL/min/1.73m² (95% confidence interval 2.0–4.4; p<0.001).

Renal outcome data revealed a notable divergence between the cohorts, underscoring the nephroprotective potency of SGLT2 inhibitor therapy (**Table 5**). None of the participants (0 of 102, 0%) in the SGLT2 inhibitor cohort experienced renal progression, defined as a sustained eGFR decline exceeding 30%, during the six-month observation period. In contrast, 6 participants (5.9%) in the control cohort met this endpoint (Fisher’s exact p=0.029; **Supplementary Table 8**). All six renal progression events were confirmed as persistent at the six-month endpoint assessment, with no spontaneous recovery to within 30% of baseline eGFR observed. The absolute risk reduction of 5.9% corresponds to a number needed to treat of 17, meaning that for every 17 diabetic kidney disease patients with DKD and wounds treated with SGLT2 inhibitors over six months, one case of renal progression would be prevented (**Figure 6**).

**Table 5:** Renal outcomes at six months.

Outcome	SGLT2 inhibitor (n=102)	Control (n=102)	Difference (95% CI)	p-value
eGFR values, mL/min/1.73m²				
Baseline	57.4 ± 14.5	54.6 ± 15.3	2.8 (−1.3 to 6.9)	0.184^a^
3 months	56.2 ± 14.2	52.1 ± 15.0	4.1 (0.1 to 8.1)	0.046^a^
6 months	55.1 ± 14.0	49.1 ± 14.8	6.0 (2.0 to 10.0)	0.003^a^
eGFR change from baseline				
Absolute change, mL/min/1.73m²	−2.3 ± 3.8	−5.5 ± 4.5	3.2 (2.0 to 4.4)	<0.001^a^
Percent change, %	−4.0 ± 6.6	−10.1 ± 8.2	6.1 (4.0 to 8.2)	<0.001^a^
Renal progression events				
Sustained ≥30% eGFR decline, n (%)	0 (0)	6 (5.9)	ARR 5.9%	0.029^b^
Number needed to treat	17	—	—	—
eGFR change by baseline stratum				
30–44 mL/min/1.73m² (n=52)	−1.8 ± 3.2	−5.8 ± 4.8	4.0 (2.0 to 6.0)	<0.001^a^
45–59 mL/min/1.73m² (n=78)	−2.4 ± 3.9	−5.4 ± 4.3	3.0 (1.4 to 4.6)	<0.001^a^
≥60 mL/min/1.73m² (n=74)	−2.5 ± 4.1	−5.3 ± 4.6	2.8 (0.9 to 4.7)	0.004^a^
p for interaction	—	—	—	0.634

ARR, absolute risk reduction; CI, confidence interval; eGFR, estimated glomerular filtration rate; SGLT2, sodium-glucose cotransporter-2.

Data are presented as mean ± standard deviation or n (%).

^a^Independent samples t-test. ^b^ Fisher’s exact test.

Renal progression was defined as sustained eGFR decline exceeding 30% from baseline, confirmed on two consecutive measurements separated by at least 28 days. All six renal progression events observed in the control cohort were confirmed as persistent at the six-month endpoint, with no spontaneous recovery observed. Individual patient details for renal progression events are provided in **Supplementary Table 8**.

**Figure 7:**
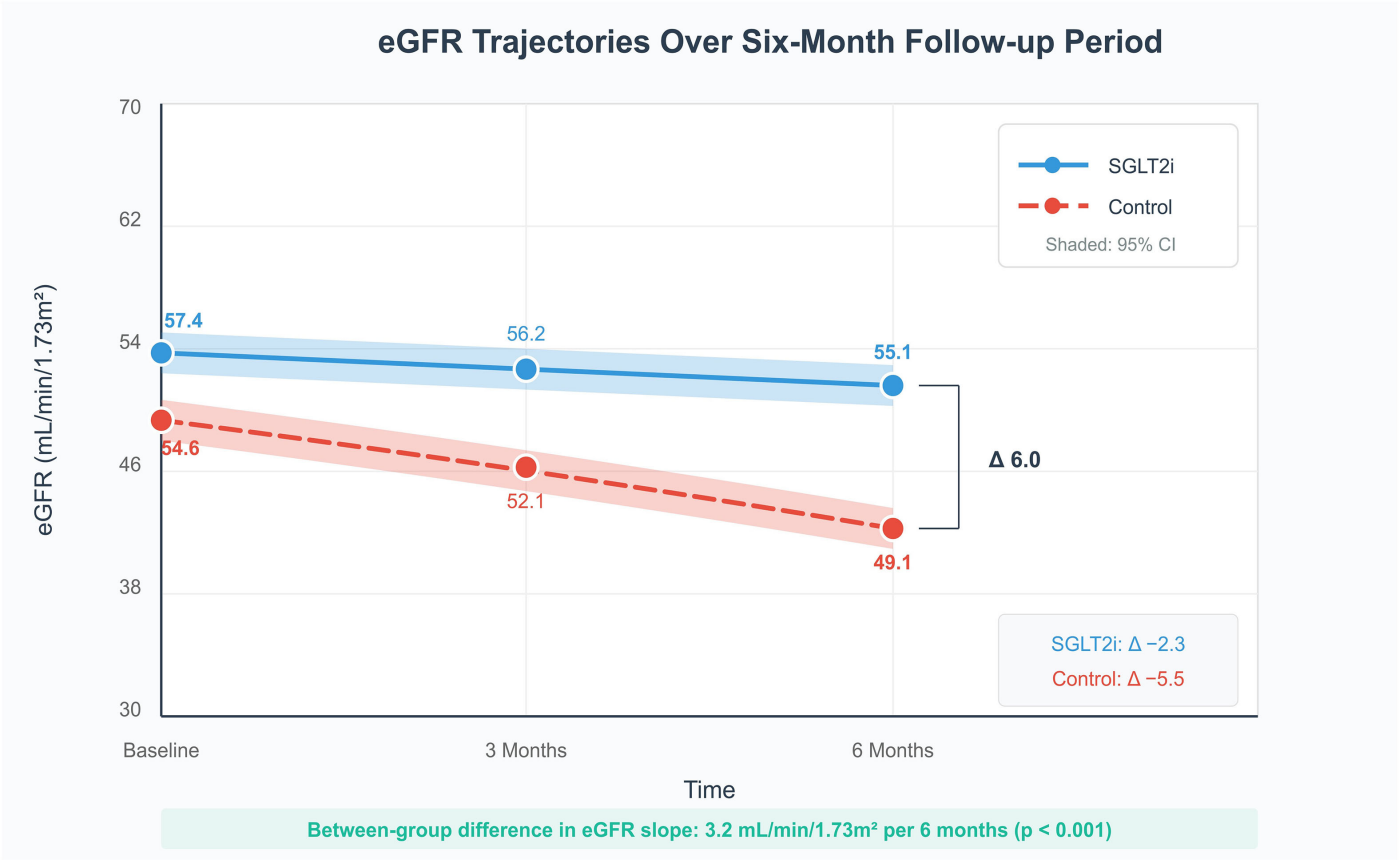
eGFR trajectories over a six-month follow-up period. A line graph depicting the mean eGFR values (mL/min/1.73m²) at baseline, three months, and six months for SGLT2 inhibitor users (solid blue line with circular markers) and controls (dashed red line with circular markers). The shaded regions represent the 95% confidence intervals. The baseline eGFR was 57.4 mL/min/1.73m² in the SGLT2 inhibitor cohort and 54.6 mL/min/1.73m ² By six months, the mean eGFR had declined to 55.1 mL/min/1.73m² (Δ −2.3) in SGLT2 inhibitor users versus 49.1 mL/min/1.73m² (Δ −5.5) in The between-group difference in the eGFR slope was 3.2 mL/min/1.73m² per six months (p<0.001). This protective effect was consistent across the baseline eGFR strata (p for interaction=0.634).

The analysis of continuous eGFR changes reinforced these findings (**Table 5**). Mean six-month eGFR change was −2.3 ± 3.8 mL/min/1.73m² in SGLT2 inhibitor users versus −5.5 ± 4.5 mL/min/1.73m² in controls, yielding a between-group difference of 3.2 mL/min/1.73m² (95% confidence interval 2.0–4.4; t-test: df=202, p<0.001). This magnitude of eGFR preservation is clinically meaningful; if sustained over longer periods, such attenuation of decline might potentially translate to delays in kidney failure onset and dialysis initiation, although this remains to be confirmed by dedicated long-term studies 30. Importantly, this protective effect was consistent across baseline eGFR strata (p for interaction=0.634), suggesting a benefit regardless of initial kidney function within the studied range (**Figure 7**).

### 3.5 Sensitivity and subgroup analyses

Sensitivity analyses consistently supported the robustness of the primary findings (**Supplementary Methods S5** and **S6**; **Supplementary Table 4**). The inverse probability of treatment weighting produced nearly identical results (adjusted hazard ratio for wound healing, 1.05; 95% confidence interval, 0.79–1.39; p=0.742). The E-value for the primary endpoint was 1.42, indicating that an unmeasured confounder would need to increase the likelihood of both SGLT2 inhibitor use and faster wound healing by at least 42% to fully explain the observed null association, a threshold exceeding the strength of most known prognostic factors, thereby suggesting reasonable robustness to residual confounding (32). Multiple imputations for the 3 participants (1.5%) with incomplete baseline data yielded virtually unchanged point estimates (**Supplementary Methods S4**).

**Figure 8:**
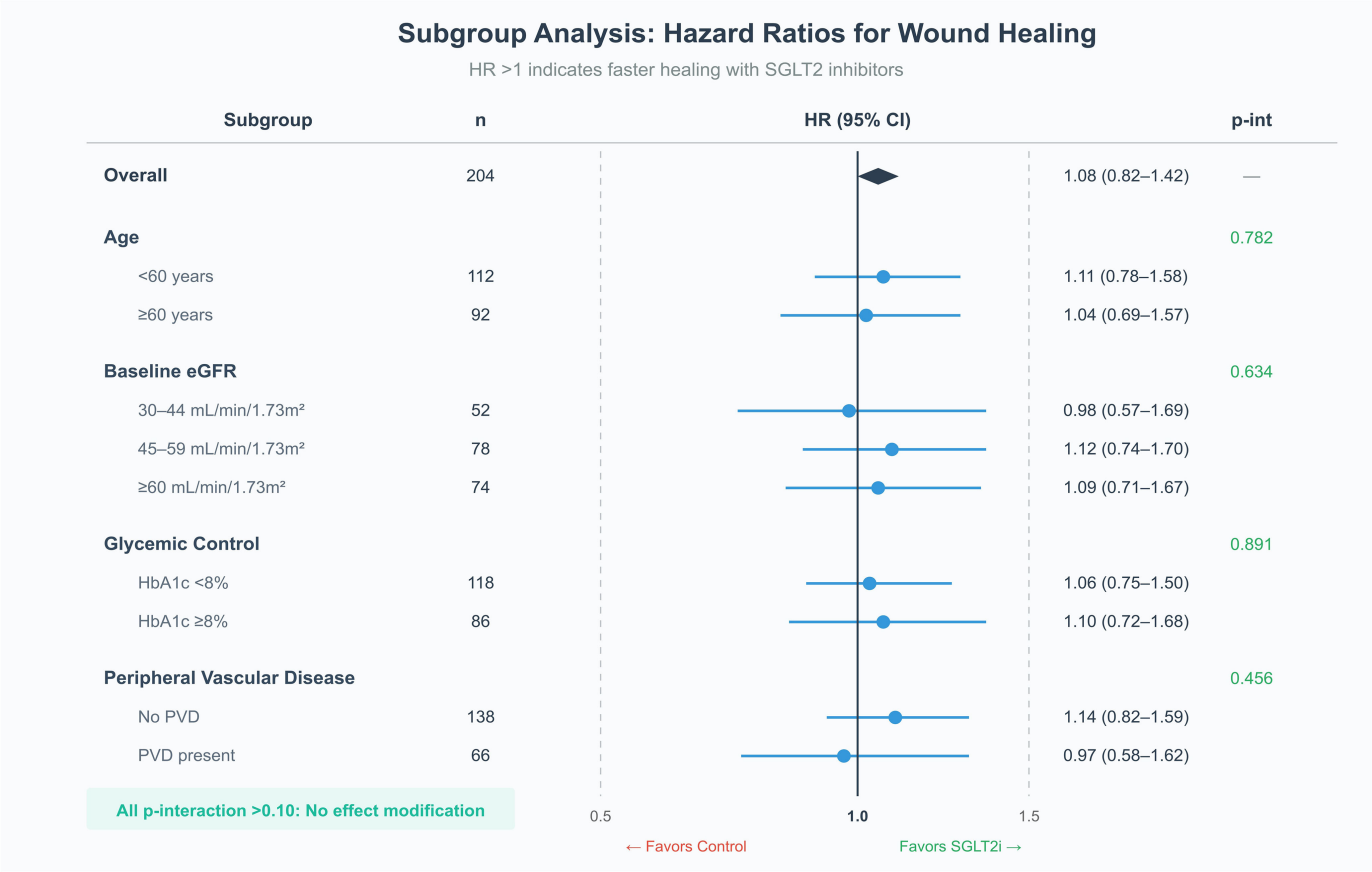
Subgroup analysis: hazard ratios for wound healing. Forest plot displaying hazard ratios with 95% confidence intervals for the time to complete wound healing across the prespecified subgroups. Hazard ratios greater than 1.0 indicate faster healing with SGLT2 inhibitor therapy. The overall effect estimate (hazard ratio 1.08, 95% confidence interval 0.82–1.42) is shown at the top of the figure. Subgroup analyses are presented for age (<60 years, n=112; ≥60 years, n=92), baseline eGFR (30–44 mL/min/1.73m², n=52; 45–59 mL/min/1.73m², n=78; ≥60 mL/min/1.73m², n=74), glycemic control (HbA1c <8%, n=118; ≥8%, n=86), and peripheral vascular disease status (absent, n=138; present, n=66). All p-values for interaction exceeded 0.10, indicating no significant effect modification across any of the examined subgroups. The vertical dashed line at a hazard ratio of 1.0 represents the null hypothesis of no difference.

Prespecified subgroup analyses revealed no significant effect modification across clinically relevant patient strata (**Figure 8**; **Supplementary Table 5**). The association between SGLT2 inhibitor use and wound healing was consistent across age categories (below vs. at or above 60 years; p for interaction=0.782), eGFR strata (30–44, 45–59, and ≥60 mL/min/1.73m²; p for interaction=0.634), glycemic control status (HbA1c below vs. at or above 8%; p for interaction=0.891), and peripheral vascular disease presence (p for interaction=0.456). When stratified by wound type (**Supplementary Table 6**), diabetic foot ulcers showed numerically greater benefit with SGLT2 inhibitor therapy (mean healing 9.2 days faster, p=0.085 unadjusted), although this observation did not survive correction for multiple comparisons and should be interpreted as hypothesis-generating rather than confirmatory.

## 4 Discussion

The central finding of this prospective cohort study is reassuring: no evidence of impaired wound healing was observed with SGLT2 inhibitor therapy in patients with diabetic kidney disease. Across every analytical approach employed (unadjusted comparison, propensity score matching, multivariable regression, inverse probability weighting), the conclusion remained consistent. Wound healing trajectories in SGLT2 inhibitor users were statistically indistinguishable from those in matched controls, with the median time to complete epithelialization differing by only one day between the cohorts. Numerical trends favored the SGLT2 inhibitor group; however, these differences were neither statistically significant nor clinically meaningful. Equally reassuring, infection rates showed no elevation with SGLT2 inhibitor use, providing no support for concerns that glycosuria-mediated immunocompromise might predispose patients to wound complications.

These null findings for wound outcomes are particularly significant when juxtaposed with the pronounced renal benefits observed. During the six-month follow-up period, no renal progression events were observed among SGLT2 inhibitor users, with not a single participant crossing the clinically important threshold of a 30% sustained eGFR decline. The magnitude of eGFR preservation (3.2 mL/min/1.73 m ² better than controls) is broadly consistent with trajectory differences reported in DAPA-CKD and EMPA-KIDNEY 4,5, suggesting that the nephroprotective effects demonstrated in carefully controlled trial populations may favorably translate to more heterogeneous patients encountered in clinical practice. In patients with DKD and an active wound, our data suggest that continuing or initiating SGLT2 inhibitor therapy may confer substantial renal benefits without any detectable wound healing penalty.

The apparent discrepancy between the numerical mean difference in healing time (4.8 days favoring SGLT2 inhibitors, p = 0.183) and the Cox hazard ratio (1.08, p = 0.594) warrants comment, as these two analytical approaches capture different aspects of the time-to-event distribution. The mean difference is sensitive to distributional skewness and outlying late-healing observations, whereas the Cox hazard ratio reflects the instantaneous healing rate across the entire follow-up period and is more robust to extreme values. The control group exhibited greater heterogeneity (standard deviation [SD]: 28.8 vs. 22.2 days), with a small proportion of participants experiencing prolonged healing beyond 90 days, which inflated the mean without substantially affecting the Kaplan–Meier curves. The hazard ratio (HR) of 1.08 indicates a trivial and non-significant 8% higher instantaneous probability of healing at any given time point, entirely consistent with the overlapping Kaplan–Meier curves (log-rank p = 0.412). Furthermore, the adjusted Cox model incorporates covariates that attenuate the unadjusted mean difference. Taken together, both analytical approaches converge on the same conclusion: no evidence of impaired wound healing was observed with SGLT2 inhibitor therapy.

The apprehension surrounding SGLT2 inhibitors and tissue integrity traces to the CANVAS Program’s amputation signal, a finding that has proven highly influential despite its ultimate non-replication (9, 10). This trial reported 6.3 amputations per 1000 patient-years with canagliflozin versus 3.4 with placebo, predominantly involving toes and metatarsals rather than major limb loss. The biological mechanism, if any, has never been identified, and subsequent megatrials enrolling tens of thousands of participants found no excess amputation risk. The CREDENCE trial, which specifically enrolled high-risk patients with diabetic kidney disease and albuminuria, reported amputation rates of 12.3 versus 11.2 per 1000 patient-years, a non-significant difference that led to the eventual removal of regulatory warnings (3, 11). A recent systematic review and meta-analysis of cardiovascular outcome trials concluded that SGLT2 inhibitors do not significantly increase the risk of amputation when data are pooled across all available trials (33). Our findings extend this reassurance to the specific clinical scenario of active wounds, a context in which many clinicians remain hesitant despite accumulating safety data.

The mechanistic basis of our null wound healing findings likely reflects a balance between the opposing pathophysiological forces. SGLT2 inhibitors induce osmotic diuresis and modest intravascular volume contraction, effects that could theoretically compromise peripheral tissue perfusion (12). However, these agents reduce oxidative stress, attenuate systemic inflammation, improve endothelial function, and may enhance autophagy, processes that collectively support rather than impede tissue repair (34, 35). The nephroprotective mechanism of SGLT2 inhibitors involves the restoration of tubuloglomerular feedback, whereby increased sodium delivery to the macula densa triggers afferent arteriolar vasoconstriction, reducing intraglomerular pressure, and hyperfiltration (36, 37). This hemodynamic effect, while protective for the glomeruli, did not appear to compromise peripheral wound healing to any detectable degree. The net effect, as suggested by our data, appears to approximate equipoise; whatever perfusion-related decrement might occur is apparently offset by favorable metabolic and inflammatory modulation.

Although propensity score matching achieved excellent covariate balance with all standardized mean differences below 0.10, peripheral vascular disease showed the largest absolute difference after matching (38.2% vs. 26.5%, SMD = 0.08). While this value remains within the conventionally accepted threshold, the clinical relevance of PVD to wound healing outcomes warrants careful consideration. Several analytical safeguards mitigate concerns about residual confounding from this source. First, PVD was explicitly included as a covariate in both the multivariable Cox and logistic regression models, providing direct adjustment for any residual imbalance. Second, the prespecified subgroup analysis stratified by PVD status revealed no significant interaction (p for interaction = 0.456), with consistent null findings for wound healing in both PVD-positive and PVD-negative subgroups. Third, the inverse probability of treatment weighting analysis, which adjusts for all 15 covariates through propensity score weights, yielded a virtually identical hazard ratio (1.05; 95% CI: 0.79–1.39). Collectively, these convergent findings provide reassurance that residual confounding from PVD is unlikely to have meaningfully distorted our primary conclusions.

Our observation that glycemic control powerfully predicts infection risk deserves clinical attention. A 1-SD elevation in HbA1c (approximately 1.1 percentage points) increased the odds of infection by nearly 50%, indicating that hyperglycemia is a dominant modifiable risk factor. This finding is consistent with the well-established relationship between glucose levels and immune function, in which hyperglycemia impairs neutrophil chemotaxis, phagocytosis, and bactericidal activity (38). The fact that effective glucose-lowering therapy is withheld from diabetic patients while they are healing wounds should not be overlooked by clinicians. Furthermore, SGLT2 inhibitors have demonstrated anti-inflammatory properties in multiple experimental models, including the reduction of inflammatory biomarkers such as interleukin-6 and tumor necrosis factor-alpha, effects that might theoretically support rather than impair wound healing (34).

Several methodological strengths enhance our confidence in our findings. The prospective design with protocolized follow-up ensured complete outcome ascertainment and standardized assessment, avoiding recall bias and incomplete data that plague retrospective investigations. Propensity score matching effectively balanced 15 measured confounders, achieving excellent covariate equilibrium that minimized confounding by indication, a critical consideration given the non-random nature of SGLT2 inhibitor prescription. The inclusion of multiple sensitivity analyses (inverse probability weighting, E-value calculation, and multiple imputation) consistently corroborated the primary results, reassuring that our conclusions are not artifacts of any single analytical approach. Independent blinded adjudication of the wound healing endpoint eliminated potential assessment bias.

We acknowledge several limitations that may affect the interpretation of our results. First, despite rigorous confounding control, residual bias from unmeasured variables cannot be excluded from any observational study. The E-value provides quantitative reassurance; however, unmeasured confounders of sufficient strength could theoretically distort our estimates. Second, although our sample size was adequate for the primary endpoint based on *a priori* power calculations, it constrained the statistical power of the subgroup analyses. The numerically faster healing observed in patients with diabetic foot ulcers treated with SGLT2 inhibitors—a difference exceeding nine days—could represent either a true subgroup effect or a chance finding; larger studies focusing specifically on this wound type would be informative. Third, the six-month observation period, although sufficient to capture wound healing and short-term renal trajectories, precludes the assessment of longer-term outcomes, including wound recurrence, sustained nephroprotection, and longer-term safety. Fourth, enrollment at a single tertiary center with specialized wound care expertise may limit the generalizability of the results to community practice settings. Finally, our study excluded patients with the most severe wounds (those with exposed bone) and the most advanced kidney disease (eGFR < 30 mL/min/1.73m²), populations in whom dedicated investigation remains warranted.

These findings have immediate clinical implications. Our data argue against the discontinuation or avoidance of reflexive SGLT2 inhibitor therapy in patients with DKD who present with wounds. The theoretical concern that motivated such practice—that glycosuria and diuresis impair wound healing— was not supported by our prospective observations. In contrast, the nephroprotective benefits are substantial and accrue rapidly; the kidney function divergence documented in this study emerged within six months. The 2024 KDIGO guidelines recommend SGLT2 inhibitors for essentially all patients with DKD disease (8), and our findings suggest that this recommendation should not be suspended in the presence of concurrent wounds.

## 5 Conclusion

In conclusion, this prospective propensity score-matched cohort study provides robust evidence that SGLT2 inhibitor therapy did not impair wound healing or increase infection risk in patients with DKD. The anticipated nephroprotective benefits were preserved during the six-month follow-up period in this clinically complex population. These findings support individualized decision-making that favors SGLT2 inhibitor continuation or initiation in appropriate candidates, regardless of wound status, while acknowledging that longer-term outcomes beyond six months require further investigation.

## Data Availability

The original contributions presented in the study are included in the article/**Supplementary Material**. Further inquiries can be directed to the corresponding authors.

## Supplementary material

Supplementary Figure 1 Propensity score distributions before and after matching. Histograms depicting the distribution of estimated propensity scores for SGLT2 inhibitor users (blue bars) and controls (red bars) before (Panel A) and after matching (Panel B). Before matching, the distributions showed limited overlap, with a C-statistic of 0.74, indicating moderate discrimination between treatment groups, reflecting systematic differences in prescribing patterns. The highlighted region in Panel A indicates the zone of limited, common support. After 1:1 nearest-neighbor matching with a caliper of 0.20 standard deviations of the propensity score logit, excellent overlap was achieved (Panel B), with nearly complete superimposition of the distributions. All matched pairs fell within the region of common support, confirming the validity of the causal inference from the matched comparisons.

Supplementary Figure 2 shows the log-log survival plots for proportional hazards assessment. Log-log transformation of cumulative survival (wound remaining unhealed) plots for the assessment of the proportional hazard assumption underlying Cox regression models. Panel A displays the primary outcome (time to complete wound healing), and Panel B displays the secondary outcome (time to renal progression). For both outcomes, the log-log survival curves for SGLT2 inhibitor users (blue lines) and controls (red lines) were approximately parallel throughout the observation period, indicating that the proportional hazards assumption was satisfied. This visual assessment was corroborated by non-significant Schoenfeld residual tests (primary outcome: χ²=0.42, p=0.517; renal progression: χ²=0.89, p=0.346), which validated the use of Cox proportional hazards regression for time-to-event analyses.

Supplementary Figure 3 Calibration plot for wound infection logistic regression model. Assessment of calibration for the multivariable logistic regression model predicting wound infection. The x-axis represents the predicted probability of wound infection based on the model; the y-axis represents the observed proportion of wound infections within each decile of predicted risk. Red circles with error bars (± standard error) indicate the observed infection rates within each risk decile. The solid blue line represents the LOESS-smoothed calibration, and the dashed gray diagonal line indicates perfect calibration (predicted = observed). The shaded blue region represents the 95% confidence band of the calibration curve. The non-significant Hosmer–Lemeshow goodness-of-fit test (χ²=6.84, df=8, p=0.554) and calibration slope near unity (0.97, 95% confidence interval 0.82–1.12) indicate excellent agreement between predicted probabilities and observed infection rates. Model discrimination was acceptable (C-statistic/AUC, 0.71; Brier score, 0.18).
