# Supplementary figures and images for: Wound healing outcomes in diabetic kidney disease patients receiving SGLT2 inhibitor therapy: a prospective propensity score-matched cohort study
Source: Front Endocrinol (Lausanne). 2026 Jun 3;17:1793030. doi: 10.3389/fendo.2026.1793030 (PMC13271949; doi:10.3389/fendo.2026.1793030)

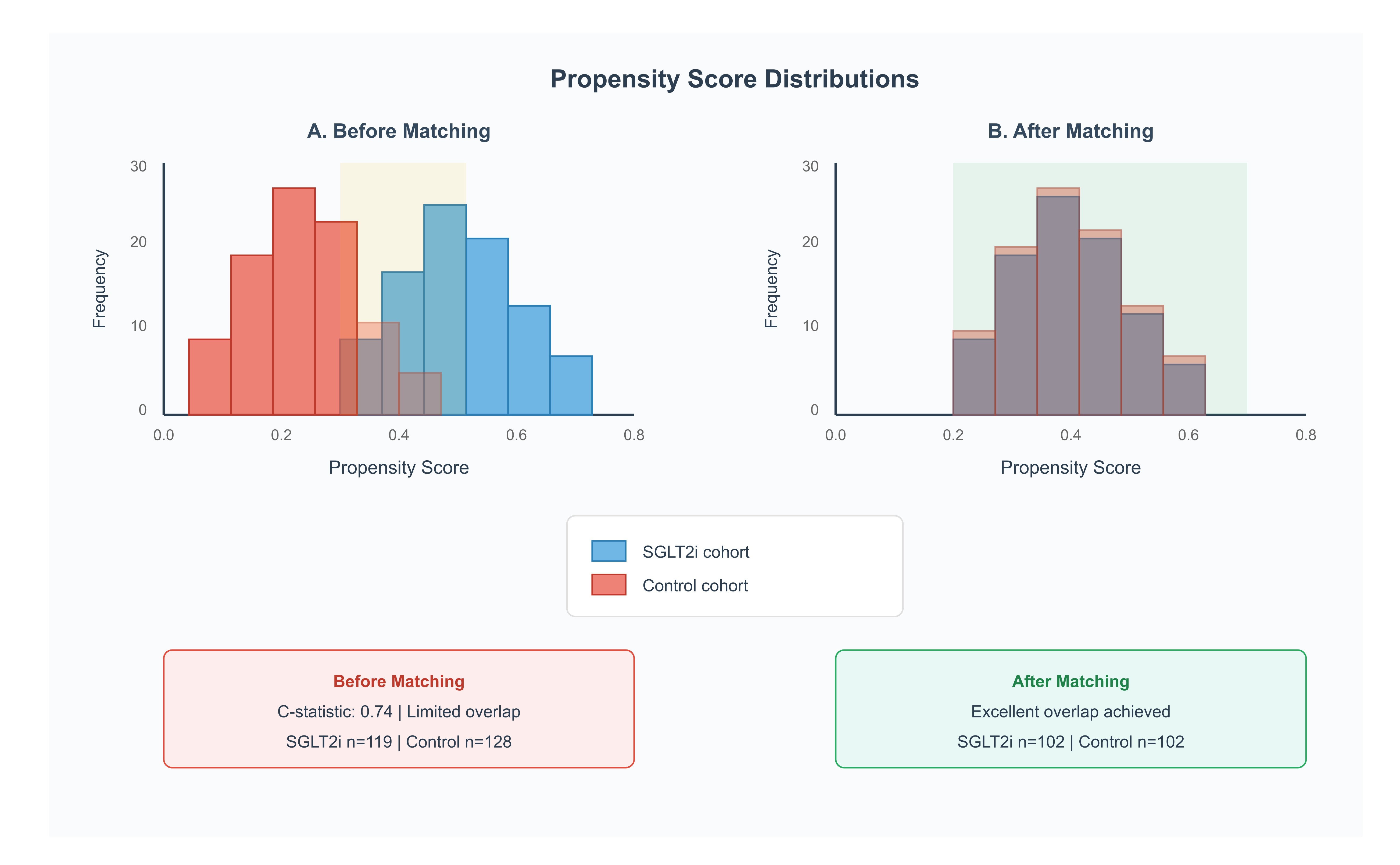

Supplement: Supplementary Figure 1 — Propensity score distributions before and after matching. Histograms depicting the distribution of estimated propensity scores for SGLT2 inhibitor users (blue bars) and controls (red bars) before (Panel A) and after matching (Panel B). Before matching, the distributions showed limited overlap, with a C-statistic of 0.74, indicating moderate discrimination between treatment groups, reflecting systematic differences in prescribing patterns. The highlighted region in Panel A indicates the zone of limited, common support. After 1:1 nearest-neighbor matching with a caliper of 0.20 standard deviations of the propensity score logit, excellent overlap was achieved (Panel B), with nearly complete superimposition of the distributions. All matched pairs fell within the region of common support, confirming the validity of the causal inference from the matched comparisons. [file Image1.jpeg]

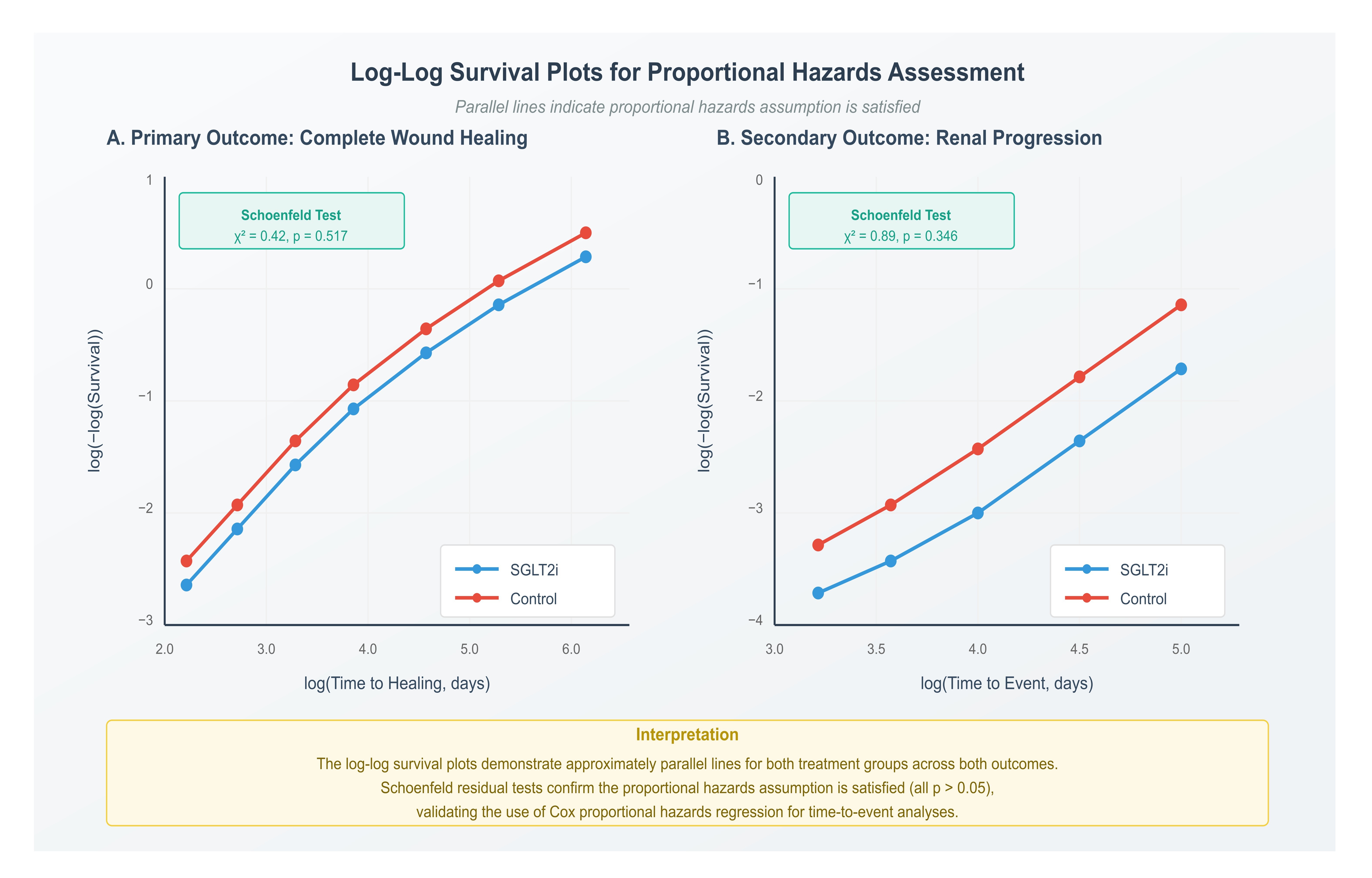

Supplement: Supplementary Figure 2 — shows the log-log survival plots for proportional hazards assessment. Log-log transformation of cumulative survival (wound remaining unhealed) plots for the assessment of the proportional hazard assumption underlying Cox regression models. Panel A displays the primary outcome (time to complete wound healing), and Panel B displays the secondary outcome (time to renal progression). For both outcomes, the log-log survival curves for SGLT2 inhibitor users (blue lines) and controls (red lines) were approximately parallel throughout the observation period, indicating that the proportional hazards assumption was satisfied. This visual assessment was corroborated by non-significant Schoenfeld residual tests (primary outcome: χ²=0.42, p=0.517; renal progression: χ²=0.89, p=0.346), which validated the use of Cox proportional hazards regression for time-to-event analyses. [file Image2.jpeg]

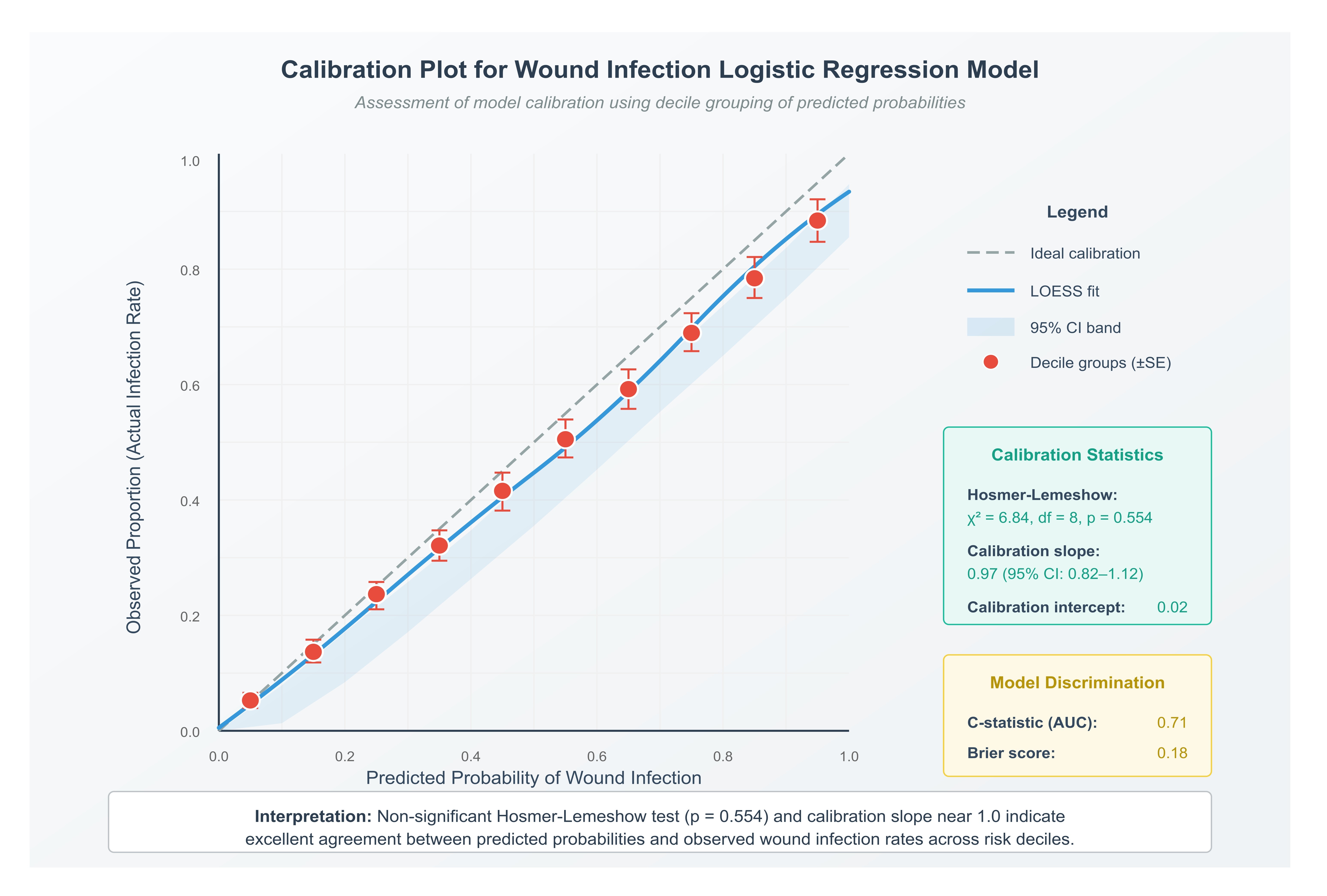

Supplement: Supplementary Figure 3 — Calibration plot for wound infection logistic regression model. Assessment of calibration for the multivariable logistic regression model predicting wound infection. The x-axis represents the predicted probability of wound infection based on the model; the y-axis represents the observed proportion of wound infections within each decile of predicted risk. Red circles with error bars (± standard error) indicate the observed infection rates within each risk decile. The solid blue line represents the LOESS-smoothed calibration, and the dashed gray diagonal line indicates perfect calibration (predicted = observed). The shaded blue region represents the 95% confidence band of the calibration curve. The non-significant Hosmer–Lemeshow goodness-of-fit test (χ²=6.84, df=8, p=0.554) and calibration slope near unity (0.97, 95% confidence interval 0.82–1.12) indicate excellent agreement between predicted probabilities and observed infection rates. Model discrimination was acceptable (C-statistic/AUC, 0.71; Brier score, 0.18). [file Image3.jpeg]
