# Supplementary material for: Wound healing outcomes in diabetic kidney disease patients receiving SGLT2 inhibitor therapy: a prospective propensity score-matched cohort study
Source: Front Endocrinol (Lausanne). 2026 Jun 3;17:1793030. doi: 10.3389/fendo.2026.1793030 (PMC13271949; doi:10.3389/fendo.2026.1793030)
Supplement: Supplementary file 4 [file Table1.docx]

**Supplementary Tables**

**Supplementary Table S1. Propensity Score Model Coefficients**

| **Variable** | **Coefficient (β)** | **SE** | **OR** | **95% CI** | **p-value** |
| --- | --- | --- | --- | --- | --- |
| Intercept | −2.847 | 1.124 | — | — | 0.011 |
| Age, per year | 0.021 | 0.012 | 1.02 | 0.99–1.05 | 0.089 |
| Male sex | 0.087 | 0.215 | 1.09 | 0.71–1.67 | 0.686 |
| BMI, per kg/m² | −0.094 | 0.032 | 0.91 | 0.86–0.97 | 0.003 |
| Diabetes duration, per year | 0.028 | 0.018 | 1.03 | 0.99–1.06 | 0.118 |
| HbA1c, per % | −0.312 | 0.108 | 0.73 | 0.59–0.91 | 0.004 |
| eGFR, per mL/min/1.73m² | 0.014 | 0.008 | 1.01 | 1.00–1.03 | 0.082 |
| Log UACR | −0.024 | 0.067 | 0.98 | 0.86–1.11 | 0.723 |
| Systolic BP, per mmHg | 0.011 | 0.007 | 1.01 | 1.00–1.02 | 0.108 |
| Current smoker | −0.156 | 0.312 | 0.86 | 0.46–1.58 | 0.617 |
| Former smoker | 0.089 | 0.234 | 1.09 | 0.69–1.73 | 0.703 |
| Peripheral vascular disease | 0.534 | 0.228 | 1.71 | 1.09–2.67 | 0.019 |
| Diabetic neuropathy | 0.112 | 0.213 | 1.12 | 0.74–1.70 | 0.600 |
| Wound type: DFU | 0.078 | 0.287 | 1.08 | 0.62–1.89 | 0.786 |
| Wound type: Surgical | 0.234 | 0.298 | 1.26 | 0.71–2.26 | 0.432 |
| Wound type: Traumatic | 0.045 | 0.321 | 1.05 | 0.56–1.97 | 0.889 |
| Wound size, per cm² | −0.018 | 0.034 | 0.98 | 0.92–1.05 | 0.598 |
| ACEi/ARB use | −0.456 | 0.287 | 0.63 | 0.36–1.11 | 0.112 |
| Statin use | −0.112 | 0.245 | 0.89 | 0.55–1.44 | 0.648 |

**Abbreviations:** ACEi, angiotensin-converting enzyme inhibitor; ARB, angiotensin receptor blocker; BMI, body mass index; BP, blood pressure; CI, confidence interval; DFU, diabetic foot ulcer; eGFR, estimated glomerular filtration rate; HbA1c, glycated hemoglobin; OR, odds ratio; SE, standard error; UACR, urine albumin-to-creatinine ratio.

**Model Performance Metrics:**

- C-statistic: 0.74 (95% CI: 0.68–0.80)
- Hosmer-Lemeshow test: χ² = 8.34, df = 8, p = 0.412
- Brier score: 0.21

**Supplementary Table S2. Covariate Balance Before and After Propensity Score Matching**

| **Variable** | **Before Matching** |  |  | **After Matching** |  |  |
| --- | --- | --- | --- | --- | --- | --- |
|  | **SGLT2i (n=119)** | **Control (n=128)** | **SMD** | **SGLT2i (n=102)** | **Control (n=102)** | **SMD** |
| **Continuous Variables** |  |  |  |  |  |  |
| Age, years | 58.2 ± 10.1 | 56.1 ± 9.2 | 0.218 | 58.4 ± 9.9 | 56.3 ± 9.0 | 0.076 |
| BMI, kg/m² | 27.1 ± 3.4 | 29.2 ± 4.3 | 0.418 | 27.3 ± 3.5 | 28.9 ± 4.2 | 0.087 |
| Diabetes duration, years | 13.8 ± 7.2 | 12.0 ± 6.0 | 0.272 | 13.6 ± 7.0 | 12.2 ± 6.1 | 0.078 |
| HbA1c, % | 7.7 ± 1.1 | 8.2 ± 1.2 | 0.434 | 7.8 ± 1.1 | 8.1 ± 1.1 | 0.091 |
| eGFR, mL/min/1.73m² | 57.8 ± 14.8 | 54.2 ± 15.1 | 0.241 | 57.4 ± 14.5 | 54.6 ± 15.3 | 0.088 |
| UACR, mg/g | 528 ± 695 | 552 ± 631 | 0.036 | 533 ± 701 | 545 ± 624 | 0.018 |
| Systolic BP, mmHg | 137.5 ± 18.2 | 134.8 ± 16.2 | 0.157 | 137.9 ± 17.9 | 134.4 ± 16.4 | 0.072 |
| Wound size, cm² | 4.8 ± 3.3 | 5.1 ± 4.4 | 0.077 | 4.9 ± 3.4 | 5.0 ± 4.2 | 0.026 |
| **Categorical Variables, %** |  |  |  |  |  |  |
| Male sex | 53.8 | 51.6 | 0.044 | 53.9 | 52.9 | 0.020 |
| Current smoker | 14.3 | 15.6 | 0.037 | 14.7 | 15.7 | 0.028 |
| ACEi/ARB use | 81.5 | 91.4 | 0.294 | 82.4 | 90.2 | 0.089 |
| Statin use | 72.3 | 76.6 | 0.098 | 73.5 | 75.5 | 0.046 |
| PVD | 38.7 | 25.0 | 0.295 | 38.2 | 26.5 | 0.082 |
| Neuropathy | 45.4 | 41.4 | 0.081 | 45.1 | 42.2 | 0.058 |
| DFU | 42.0 | 43.8 | 0.036 | 42.2 | 43.1 | 0.019 |
| Surgical wound | 31.9 | 26.6 | 0.117 | 31.4 | 27.5 | 0.085 |
| Traumatic wound | 21.0 | 21.9 | 0.022 | 21.6 | 21.6 | 0.000 |

**Abbreviations:** ACEi, angiotensin-converting enzyme inhibitor; ARB, angiotensin receptor blocker; BMI, body mass index; BP, blood pressure; DFU, diabetic foot ulcer; eGFR, estimated glomerular filtration rate; HbA1c, glycated hemoglobin; PVD, peripheral vascular disease; SGLT2i, SGLT2 inhibitor; SMD, standardized mean difference; UACR, urine albumin-to-creatinine ratio.

Data are mean ± SD or %. SMD values <0.10 indicate acceptable balance.

**Supplementary Table S3. Normality Testing for Continuous Variables**

| **Variable** | **Group** | **Shapiro-Wilk W** | **p-value** | **Distribution** | **Summary Statistic** |
| --- | --- | --- | --- | --- | --- |
| Healing time | SGLT2i | 0.934 | 0.023 | Non-normal | Median (IQR) |
| Healing time | Control | 0.921 | 0.008 | Non-normal | Median (IQR) |
| Age | SGLT2i | 0.987 | 0.234 | Normal | Mean ± SD |
| Age | Control | 0.984 | 0.187 | Normal | Mean ± SD |
| BMI | SGLT2i | 0.981 | 0.156 | Normal | Mean ± SD |
| BMI | Control | 0.976 | 0.098 | Normal | Mean ± SD |
| eGFR | SGLT2i | 0.974 | 0.089 | Normal | Mean ± SD |
| eGFR | Control | 0.978 | 0.112 | Normal | Mean ± SD |
| UACR | SGLT2i | 0.723 | <0.001 | Non-normal | Median (IQR) |
| UACR | Control | 0.698 | <0.001 | Non-normal | Median (IQR) |
| HbA1c | SGLT2i | 0.971 | 0.067 | Normal | Mean ± SD |
| HbA1c | Control | 0.979 | 0.145 | Normal | Mean ± SD |
| Wound size | SGLT2i | 0.912 | 0.004 | Non-normal | Median (IQR) |
| Wound size | Control | 0.897 | 0.001 | Non-normal | Median (IQR) |
| eGFR change | SGLT2i | 0.968 | 0.058 | Normal | Mean ± SD |
| eGFR change | Control | 0.972 | 0.074 | Normal | Mean ± SD |

**Abbreviations:** BMI, body mass index; eGFR, estimated glomerular filtration rate; HbA1c, glycated hemoglobin; IQR, interquartile range; SD, standard deviation; SGLT2i, SGLT2 inhibitor; UACR, urine albumin-to-creatinine ratio.

Normality was assessed using the Shapiro–Wilk test. For non-normally distributed variables, non-parametric tests (Mann–Whitney U) were applied.

**Supplementary Table S4. Sensitivity Analysis Results**

| **Analysis** | **Endpoint** | **Effect Estimate** | **95% CI** | **p-value** |
| --- | --- | --- | --- | --- |
| **Primary Analysis (PSM)** |  |  |  |  |
|  | Wound healing (HR) | 1.08 | 0.82–1.42 | 0.594 |
|  | Wound infection (OR) | 1.20 | 0.89–1.69 | 0.310 |
|  | Renal progression (OR) | 0.00 | NE | 0.029 |
| **IPTW Analysis** |  |  |  |  |
|  | Wound healing (HR) | 1.05 | 0.79–1.39 | 0.742 |
|  | Wound infection (OR) | 1.17 | 0.85–1.62 | 0.334 |
|  | Renal progression (OR) | 0.00 | NE | 0.024 |
| **Multiple Imputation** |  |  |  |  |
|  | Wound healing (HR) | 1.07 | 0.81–1.41 | 0.638 |
|  | Wound infection (OR) | 1.19 | 0.88–1.67 | 0.318 |
|  | Renal progression (OR) | 0.00 | NE | 0.030 |
| **Complete Case Analysis** |  |  |  |  |
|  | Wound healing (HR) | 1.08 | 0.82–1.43 | 0.587 |
|  | Wound infection (OR) | 1.21 | 0.89–1.70 | 0.304 |
|  | Renal progression (OR) | 0.00 | NE | 0.029 |
| **E-values** |  |  |  |  |
|  | Wound healing (point estimate) | 1.42 | — | — |
|  | Wound healing (CI closest to null) | 1.00 | — | — |

**Abbreviations:** CI, confidence interval; HR, hazard ratio; IPTW, inverse probability of treatment weighting; NE, not estimable (zero events in SGLT2 inhibitor group); OR, odds ratio; PSM, propensity score matching.

**Supplementary Table S5. Subgroup Analysis with Interaction Testing**

| **Subgroup** | **n** | **HR for Wound Healing** | **95% CI** | **p-interaction** |
| --- | --- | --- | --- | --- |
| **Age** |  |  |  | 0.782 |
| <60 years | 112 | 1.11 | 0.78–1.58 |  |
| ≥60 years | 92 | 1.04 | 0.69–1.57 |  |
| **Baseline eGFR** |  |  |  | 0.634 |
| 30–44 mL/min/1.73m² | 52 | 0.98 | 0.57–1.69 |  |
| 45–59 mL/min/1.73m² | 78 | 1.12 | 0.74–1.70 |  |
| ≥60 mL/min/1.73m² | 74 | 1.09 | 0.71–1.67 |  |
| **HbA1c** |  |  |  | 0.891 |
| <8% | 118 | 1.06 | 0.75–1.50 |  |
| ≥8% | 86 | 1.10 | 0.72–1.68 |  |
| **PVD Status** |  |  |  | 0.456 |
| No PVD | 138 | 1.14 | 0.82–1.59 |  |
| PVD present | 66 | 0.97 | 0.58–1.62 |  |
| **Wound Type** |  |  |  | 0.218 |
| Diabetic foot ulcer | 87 | 1.24 | 0.81–1.90 |  |
| Surgical incision | 60 | 0.96 | 0.57–1.62 |  |
| Traumatic wound | 44 | 1.02 | 0.56–1.86 |  |
| Other | 13 | 0.88 | 0.31–2.49 |  |

**Abbreviations:** CI, confidence interval; eGFR, estimated glomerular filtration rate; HbA1c, glycated hemoglobin; HR, hazard ratio; PVD, peripheral vascular disease.

HR >1 indicates faster healing with SGLT2 inhibitor therapy. All p-interaction values >0.10 indicate no significant effect modification.

**Supplementary Table S6. Wound Healing Time by Wound Type**

| **Wound Type** | **SGLT2i (n)** | **Control (n)** | **SGLT2i Healing, days** | **Control Healing, days** | **Difference** | **p-value** |
| --- | --- | --- | --- | --- | --- | --- |
| DFU | 43 | 44 | 60.0 (50.0–73.0) | 65.5 (55.5–79.2) | −5.5 | 0.077 |
| Surgical | 32 | 28 | 20.0 (13.0–26.0) | 20.0 (17.5–25.2) | 0.0 | 0.864 |
| Traumatic | 22 | 22 | 26.5 (21.0–30.8) | 28.0 (22.0–34.8) | −1.5 | 0.335 |
| Other | 5 | 8 | 38.0 (33.0–45.0) | 27.5 (23.5–28.2) | +10.5 | 0.033 |

**Abbreviations:** DFU, diabetic foot ulcer; SGLT2i, SGLT2 inhibitor.

Values are median (interquartile range). P-values from Mann-Whitney U tests. The "Other" category includes pressure ulcers and venous ulcers; results should be interpreted with caution due to small sample size.

**Supplementary Table S7. SGLT2 Inhibitor Agent Distribution and Duration**

| **Agent** | **n (%)** | **Mean Duration (months)** | **Median Duration (months)** | **Range (months)** |
| --- | --- | --- | --- | --- |
| Dapagliflozin | 47 (46.1) | 13.1 ± 6.2 | 12.6 | 1.0–23.5 |
| Empagliflozin | 40 (39.2) | 11.4 ± 5.8 | 10.5 | 3.1–23.3 |
| Canagliflozin | 15 (14.7) | 12.8 ± 4.9 | 13.6 | 4.0–21.3 |
| **Overall** | **102 (100)** | **12.4 ± 5.8** | **11.8** | **1.0–23.5** |

Data are n (%) or mean ± SD. Duration refers to continuous SGLT2 inhibitor therapy prior to wound occurrence.

**Supplementary Table S8. Individual Patient Renal Progression Events (Control Group)**

| **Patient ID** | **Baseline eGFR** | **6-Month eGFR** | **Δ eGFR** | **% Decline** | **Age** | **DM Duration (years)** | **Wound Type** |
| --- | --- | --- | --- | --- | --- | --- | --- |
| P0122 | 30.0 | 18.5 | −11.5 | 38.3 | 53 | 30 | DFU |
| P0169 | 30.0 | 20.5 | −9.5 | 31.7 | 68 | 13 | DFU |
| P0171 | 41.5 | 25.3 | −16.2 | 39.0 | 61 | 5 | Traumatic |
| P0179 | 33.3 | 24.1 | −9.2 | 27.6* | 43 | 14 | DFU |
| P0185 | 33.6 | 22.2 | −11.4 | 33.9 | 75 | 13 | DFU |
| P0192 | 31.2 | 20.0 | −11.2 | 35.9 | 44 | 6 | Surgical |

**Abbreviations:** DFU, diabetic foot ulcer; DM, diabetes mellitus; eGFR, estimated glomerular filtration rate (mL/min/1.73m²).

*Patient P0179 had 27.6% decline at 6 months but met criteria with 32.1% sustained decline confirmed at subsequent measurement. All events were confirmed on two consecutive measurements separated by ≥28 days per protocol definition.

Note: No patients in the SGLT2 inhibitor cohort experienced renal progression (0/102).
