# Supplementary material for: Wound healing outcomes in diabetic kidney disease patients receiving SGLT2 inhibitor therapy: a prospective propensity score-matched cohort study
Source: Front Endocrinol (Lausanne). 2026 Jun 3;17:1793030. doi: 10.3389/fendo.2026.1793030 (PMC13271949; doi:10.3389/fendo.2026.1793030)
Supplement: Supplementary file 5 [file DataSheet1.docx]

**Supplementary Methods**

**S1. Detailed Propensity Score Model Specification**

The propensity score, defined as the conditional probability of receiving SGLT2 inhibitor therapy given observed baseline characteristics, was estimated using multivariable logistic regression. The model included 15 covariates selected a priori based on clinical relevance and demonstrated associations with both treatment selection and wound healing outcomes.

**S1.1 Model Formula**

The propensity score model was specified as follows:

| logit(P[SGLT2i = 1]) = β₀ + β₁(Age) + β₂(Sex) + β₃(BMI) + β₄(DM_duration) +  β₅(HbA1c) + β₆(eGFR) + β₇(log_UACR) + β₈(SBP) +  β₉(Smoking_current) + β₁₀(Smoking_former) + β₁₁(PVD) +  β₁₂(Neuropathy) + β₁₃(Wound_DFU) + β₁₄(Wound_surgical) +  β₁₅(Wound_traumatic) + β₁₆(Wound_size) + β₁₇(ACEi_ARB) +  β₁₈(Statin) |
| --- |

Where:

- SGLT2i = 1 indicates SGLT2 inhibitor use; 0 indicates control
- Age = age in years (continuous)
- Sex = male sex (binary: 1 = male, 0 = female)
- BMI = body mass index in kg/m² (continuous)
- DM_duration = diabetes duration in years (continuous)
- HbA1c = glycated hemoglobin in % (continuous)
- eGFR = estimated glomerular filtration rate in mL/min/1.73m² (continuous)
- log_UACR = natural log-transformed urine albumin-to-creatinine ratio (continuous)
- SBP = systolic blood pressure in mmHg (continuous)
- Smoking_current = current smoker (binary)
- Smoking_former = former smoker (binary; reference: never smoker)
- PVD = peripheral vascular disease (binary)
- Neuropathy = diabetic peripheral neuropathy (binary)
- Wound_DFU = diabetic foot ulcer (binary)
- Wound_surgical = surgical incision (binary)
- Wound_traumatic = traumatic wound (binary; reference: other wound type)
- Wound_size = wound area in cm² (continuous)
- ACEi_ARB = angiotensin-converting enzyme inhibitor or angiotensin receptor blocker use (binary)
- Statin = statin use (binary)

**S1.2 Model Performance Metrics**

The propensity score model was evaluated using multiple performance metrics:

| **Metric** | **Value** | **Interpretation** |
| --- | --- | --- |
| C-statistic (AUC) | 0.74 (95% CI: 0.68–0.80) | Good discrimination |
| Hosmer-Lemeshow χ² | 8.34 (df = 8, p = 0.412) | Adequate calibration |
| Brier score | 0.21 | Acceptable prediction accuracy |
| Pseudo-R² (Nagelkerke) | 0.18 | Moderate explanatory power |

The C-statistic of 0.74 indicates good discriminatory capacity—sufficient to identify systematic differences in prescribing patterns while not so high as to preclude adequate overlap between treatment groups. Full model coefficients are presented in Supplementary Table S1.

**S1.3 Covariate Selection Rationale**

Covariates were selected based on the following criteria:

1. **Clinical relevance**: Variables known or hypothesized to influence SGLT2 inhibitor prescribing decisions in clinical practice
2. **Prognostic importance**: Variables with established associations with wound healing outcomes
3. **Avoiding instruments**: Exclusion of variables that influence treatment but not outcome (which would increase variance without reducing bias)
4. **Data availability**: Variables routinely collected during clinical encounters

We did not include post-baseline variables or variables on the causal pathway between SGLT2 inhibitor exposure and wound healing to avoid overadjustment bias.

**S2. Matching Algorithm Details**

**S2.1 Matching Specifications**

| **Parameter** | **Specification** |
| --- | --- |
| Matching method | 1:1 nearest-neighbor matching |
| Replacement | Without replacement |
| Distance metric | Logit of propensity score |
| Caliper width | 0.20 × SD of propensity score logit = 0.041 |
| Common support | Enforced; participants outside region of overlap excluded |
| Matching order | Random (to avoid order effects) |

**S2.2 Caliper Width Justification**

The caliper width of 0.20 standard deviations of the propensity score logit was selected based on empirical evidence demonstrating that this threshold eliminates approximately 98% of bias from measured confounders while maintaining adequate sample size [Austin PC. Pharm Stat. 2011;10(2):150-161]. This balance between bias reduction and precision preservation is critical in observational studies with moderate sample sizes.

**S2.3 Balance Assessment**

Covariate balance was assessed using standardized mean differences (SMD), calculated as:

| SMD = (X̄_treated - X̄_control) / √[(S²_treated + S²_control) / 2]  For binary variables:  SMD = (P_treated - P_control) / √[(P_treated(1-P_treated) + P_control(1-P_control)) / 2]  The threshold of \|SMD\| < 0.10 was used to indicate negligible imbalance, consistent with published recommendations [Austin PC. Stat Med. 2009;28(25):3083-3107]. All 15 covariates achieved SMD < 0.10 following matching (Supplementary Table S2, Figure 2). |
| --- |

**S2.4 Common Support Evaluation**

Propensity score distributions were examined visually using overlapping histograms (Supplementary Figure S1) to ensure adequate common support—the region of propensity score values where both treated and control participants exist. Participants with propensity scores outside the region of common support were excluded from matching to avoid extrapolation beyond the observed data.

Before matching:

- SGLT2 inhibitor cohort: n = 119, propensity score range 0.12–0.71
- Control cohort: n = 128, propensity score range 0.08–0.62
- Region of common support: 0.12–0.62

After matching:

- Both cohorts: n = 102 each
- All matched pairs within common support region

**S3. Assessment of Proportional Hazards Assumption**

The validity of Cox proportional hazards regression depends on the assumption that the hazard ratio remains constant over time. We evaluated this assumption using complementary approaches.

**S3.1 Schoenfeld Residuals Test**

Schoenfeld residuals were calculated for each covariate and tested for correlation with time. A significant correlation (p < 0.05) would indicate violation of the proportional hazards assumption.

| **Variable** | **Correlation (ρ)** | **χ²** | **p-value** |
| --- | --- | --- | --- |
| SGLT2 inhibitor use | 0.023 | 0.06 | 0.812 |
| Wound size | −0.089 | 0.89 | 0.345 |
| Glycated hemoglobin | 0.054 | 0.33 | 0.567 |
| Peripheral vascular disease | −0.076 | 0.64 | 0.423 |
| **Global test** | — | **2.34** | **0.673** |

The Schoenfeld residual test for SGLT2 inhibitor use was non-significant (χ² = 0.42, p = 0.517), indicating no evidence of violation of the proportional hazards assumption.

**S3.2 Log-Log Survival Plots**

Visual inspection of log(-log(survival)) versus log(time) plots provides a graphical assessment of proportionality. Under the proportional hazards assumption, curves for different covariate strata should be approximately parallel.

Log-log plots for SGLT2 inhibitor use (Supplementary Figure S2, Panel A) demonstrated parallel curves throughout the observation period, corroborating the Schoenfeld residuals test results.

**S3.3 Time-Varying Covariate Analysis**

As an additional sensitivity analysis, we fitted an extended Cox model including an interaction term between SGLT2 inhibitor use and log(time):

h(t) = h₀(t) × exp(β₁×SGLT2i + β₂×SGLT2i×log(t) + β₃×Covariates)

The interaction term was non-significant (β₂ = 0.02, SE = 0.08, p = 0.78), providing further evidence that the treatment effect does not vary meaningfully over time.

**S4. Multiple Imputation Procedure**

Three participants (1.5%) had incomplete baseline covariate data. To address potential bias from complete-case analysis, we employed multiple imputation.

**S4.1 Missing Data Pattern**

| **Variable** | **n Missing** | **% Missing** |
| --- | --- | --- |
| HbA1c | 2 | 0.8% |
| UACR | 1 | 0.4% |
| All other variables | 0 | 0% |

The missing data pattern was consistent with the missing at random (MAR) assumption, as missingness was associated with administrative factors (laboratory results not available within the 90-day window) rather than the values themselves.

**S4.2 Imputation Model Specifications**

| **Parameter** | **Specification** |
| --- | --- |
| Method | Multiple imputation by chained equations (MICE) |
| Number of imputations | 20 |
| Imputation model for continuous variables | Predictive mean matching |
| Imputation model for binary variables | Logistic regression |
| Variables included | All analysis variables |
| Auxiliary variables | Smoking pack-years, wound duration at presentation |
| Maximum iterations | 20 |
| Convergence criterion | Assessed by trace plots |

**S4.3 Imputation Model Details**

For continuous variables (HbA1c, UACR), predictive mean matching was used to preserve the distributional properties of the observed data. This approach imputes missing values by selecting observed values from donors with similar predicted values, avoiding the generation of implausible imputed values.

The imputation model included:

- All variables in the analysis model (to satisfy the congeniality requirement)
- Auxiliary variables correlated with the missing variables or missingness (to improve imputation accuracy and reduce bias)
- The outcome variable (to preserve the outcome-covariate relationship)

**S4.4 Convergence Assessment**

Convergence of the MICE algorithm was assessed by examining trace plots of the mean and standard deviation of imputed values across iterations. All chains demonstrated convergence by iteration 10, well within the 20-iteration maximum.

**S4.5 Analysis of Imputed Datasets**

Following Rubin's rules, parameter estimates and standard errors were combined across the 20 imputed datasets:

**Combined estimate:**

β̄ = (1/m) × Σβ̂ⱼ

**Combined variance:**

Var(β̄) = W̄ + (1 + 1/m) × B

Where:

- m = number of imputations (20)
- W̄ = within-imputation variance (mean of variance estimates)
- B = between-imputation variance (variance of point estimates)

Results from multiple imputation were virtually identical to complete-case analysis (Supplementary Table S4), reflecting the minimal amount of missing data.

**S5. E-Value Calculation**

The E-value quantifies the minimum strength of association that an unmeasured confounder would need to have with both the treatment and the outcome to fully explain away an observed treatment-outcome association, conditional on the measured covariates.

**S5.1 E-Value Formula**

For hazard ratios, the E-value is calculated as:

E-value = HR + √(HR × (HR - 1))

For the confidence interval limit closest to the null:

E-value_CI = HR_CI + √(HR_CI × (HR_CI - 1))

Where HR is expressed as a value ≥ 1 (if HR < 1, use 1/HR).

**S5.2 E-Value Results**

**Primary endpoint (wound healing):**

| **Measure** | **Value** | **E-value** | **Interpretation** |
| --- | --- | --- | --- |
| Point estimate | HR = 1.08 | 1.42 | An unmeasured confounder would need RR ≥ 1.42 with both treatment and outcome |
| 95% CI lower bound | HR = 0.82 | 1.00 | The CI includes the null; E-value equals 1.00 |
| 95% CI upper bound | HR = 1.42 | 2.21 | — |

**S5.3 Interpretation**

The E-value of 1.42 for the point estimate indicates that an unmeasured confounder associated with both SGLT2 inhibitor use and wound healing by risk ratios of at least 1.42 each could explain away the observed association. This threshold exceeds the strength of association of most known wound healing predictors with the exception of wound size.

For context, the strongest measured confounder in our analysis (wound size) had an association with wound healing of approximately HR = 0.95 per cm² (or HR ≈ 1.05 for smaller wounds). An unmeasured confounder would need to be substantially stronger than wound size to fully explain our findings.

The E-value for the confidence interval limit closest to the null is 1.00, indicating that the confidence interval already includes values consistent with no unmeasured confounding effect. This reflects the non-significant p-value for the primary endpoint.

**S5.4 Limitations of E-Value Interpretation**

The E-value provides a quantitative benchmark for assessing robustness to unmeasured confounding but has limitations:

1. It assumes a single unmeasured confounder; multiple weaker confounders could jointly explain the association
2. It does not account for measurement error in measured confounders
3. It provides an upper bound on the strength of confounding required, not evidence that such confounding exists or does not exist

**S6. Inverse Probability of Treatment Weighting**

As an alternative to propensity score matching, we conducted inverse probability of treatment weighting (IPTW) analysis to assess the robustness of findings.

**S6.1 Weight Calculation**

IPTW creates a pseudo-population in which treatment assignment is independent of measured confounders. Weights were calculated as:

**For SGLT2 inhibitor users:**

w = 1 / PS

**For controls:**

w = 1 / (1 - PS)

Where PS = propensity score.

**S6.2 Weight Stabilization**

To reduce variance from extreme weights, we employed stabilized weights:

**For SGLT2 inhibitor users:**

sw = P(SGLT2i = 1) / PS

**For controls:**

sw = P(SGLT2i = 0) / (1 - PS)

Where P(SGLT2i = 1) and P(SGLT2i = 0) are the marginal probabilities of treatment.

**S6.3 Weight Distribution**

| **Statistic** | **Unstabilized Weights** | **Stabilized Weights** |
| --- | --- | --- |
| Mean | 2.08 | 1.00 |
| Median | 1.85 | 0.94 |
| Range | 1.12–8.94 | 0.54–4.31 |
| % Weights > 10 | 0% | 0% |

The stabilized weights had a mean of 1.00 (as expected) with no extreme values, indicating appropriate weight distribution.

**S6.4 IPTW Analysis Results**

Weighted Cox regression and logistic regression models were fitted using the stabilized weights. Robust (sandwich) standard errors were used to account for the weighting. Results were consistent with the primary propensity score matching analysis (Supplementary Table S4).

**S7. Statistical Software and Reproducibility**

**S7.1 Software Specifications**

All analyses were performed using Python version 3.10.8 with the following packages:

| **Package** | **Version** | **Purpose** |
| --- | --- | --- |
| scipy | 1.9.3 | Statistical tests |
| statsmodels | 0.13.5 | Regression models |
| lifelines | 0.27.4 | Survival analysis |
| numpy | 1.23.5 | Numerical operations |
| pandas | 1.5.2 | Data manipulation |
| scikit-learn | 1.2.0 | Propensity score estimation |
| matplotlib | 3.6.2 | Visualization |
| seaborn | 0.12.1 | Statistical visualization |

**S7.2 Random Seed**

For reproducibility of stochastic procedures (propensity score matching order, multiple imputation), random seed was set to 42 at the beginning of each analysis script.

**S7.3 Code Availability**

Analysis code is available from the corresponding author upon reasonable request.

**References for Supplementary Methods**

1. Austin PC. Optimal caliper widths for propensity-score matching when estimating differences in means and differences in proportions in observational studies. Pharm Stat. 2011;10(2):150-161.
2. Austin PC. Balance diagnostics for comparing the distribution of baseline covariates between treatment groups in propensity-score matched samples. Stat Med. 2009;28(25):3083-3107.
3. Grambsch PM, Therneau TM. Proportional hazards tests and diagnostics based on weighted residuals. Biometrika. 1994;81(3):515-526.
4. van Buuren S, Groothuis-Oudshoorn K. mice: Multivariate imputation by chained equations in R. J Stat Softw. 2011;45(3):1-67.
5. Rubin DB. Multiple Imputation for Nonresponse in Surveys. New York: John Wiley & Sons; 1987.
6. VanderWeele TJ, Ding P. Sensitivity analysis in observational research: introducing the E-value. Ann Intern Med. 2017;167(4):268-274.
7. Robins JM, Hernán MA, Brumback B. Marginal structural models and causal inference in epidemiology. Epidemiology. 2000;11(5):550-560.
8. Cole SR, Hernán MA. Constructing inverse probability weights for marginal structural models. Am J Epidemiol. 2008;168(6):656-664.
